# Non-invasive Imaging of Sendai Virus Infection in Pharmacologically Immunocompromised Mice: NK and T Cells, but not Neutrophils, Promote Viral Clearance after Therapy with Cyclophosphamide and Dexamethasone

**DOI:** 10.1371/journal.ppat.1005875

**Published:** 2016-09-02

**Authors:** Heba H. Mostafa, Peter Vogel, Ashok Srinivasan, Charles J. Russell

**Affiliations:** 1 Department of Infectious Diseases, St. Jude Children’s Research Hospital, Memphis, Tennessee, United States of America; 2 Department of Pathology, St. Jude Children’s Research Hospital, Memphis, Tennessee, United States of America; 3 Department of Bone Marrow Transplantation and Cellular Therapy, St. Jude Children’s Research Hospital, Memphis, Tennessee, United States of America; 4 Department of Pediatrics, College of Medicine, University of Tennessee Health Science Center, Memphis, Tennessee, United States of America; 5 Department of Microbiology, Immunology & Biochemistry, College of Medicine, University of Tennessee Health Science Center, Memphis, Tennessee, United States of America; National Institutes of Health, UNITED STATES

## Abstract

In immunocompromised patients, parainfluenza virus (PIV) infections have an increased potential to spread to the lower respiratory tract (LRT), resulting in increased morbidity and mortality. Understanding the immunologic defects that facilitate viral spread to the LRT will help in developing better management protocols. In this study, we immunosuppressed mice with dexamethasone and/or cyclophosphamide then monitored the spread of viral infection into the LRT by using a noninvasive bioluminescence imaging system and a reporter Sendai virus (murine PIV type 1). Our results show that immunosuppression led to delayed viral clearance and increased viral loads in the lungs. After cessation of cyclophosphamide treatment, viral clearance occurred before the generation of Sendai-specific antibody responses and coincided with rebounds in neutrophils, T lymphocytes, and natural killer (NK) cells. Neutrophil suppression using anti-Ly6G antibody had no effect on infection clearance, NK-cell suppression using anti-NK antibody delayed clearance, and T-cell suppression using anti-CD3 antibody resulted in no clearance (chronic infection). Therapeutic use of hematopoietic growth factors G-CSF and GM-CSF had no effect on clearance of infection. In contrast, treatment with Sendai virus—specific polysera or a monoclonal antibody limited viral spread into the lungs and accelerated clearance. Overall, noninvasive bioluminescence was shown to be a useful tool to study respiratory viral progression, revealing roles for NK and T cells, but not neutrophils, in Sendai virus clearance after treatment with dexamethasone and cyclophosphamide. Virus-specific antibodies appear to have therapeutic potential.

## Author Summary

Parainfluenza viruses (PIV) are major respiratory pathogens that infect almost all children before the age of 5. Infection is particularly severe and life threatening in immunocompromised patients. Although infections among immunocompromised patients are common, there are currently no effective therapeutic or preventive measures. Here, we studied the progression of PIV infection in living, immunocompromised mice by non-invasive bioluminescence imaging. We also tested the outcome of treating infected, immunocompromised mice with various intervention approaches designed to target the virus by specific antibodies or modulating the host’s immune system using drugs that increase neutrophils or B- and T-cells broadly. Bioluminescence imaging was demonstrated to quantify respiratory infection with greater precision, less variability, and fewer animals than classic techniques that require euthanizing groups of animals at defined time points. Non-invasive imaging of infection in immunocompromised hosts is also shown to be well suited to track sustained infections and develop novel preventive and therapeutic strategies.

## Introduction

Paramyxoviruses are responsible for approximately half of all respiratory viral—related hospitalizations of children in the United States [1], and they cause high levels of morbidity and mortality in immunocompromised patients of any age [2, 3]. These viruses include human respiratory syncytial virus (RSV), human metapneumovirus, and the human parainfluenza viruses (HPIVs). This study focuses on the PIVs. HPIV1 is the leading cause of severe croup (laryngotracheobronchitis), and HPIV3 is a leading cause of bronchiolitis and pneumonia [4–6]. There are no licensed vaccines or other therapeutic agents against these viruses, yet nearly all children become infected with them by the age of 5 years [7].

In immunocompromised patients, HPIV infections last longer and have more LRT involvement than do infections in immunocompetent individuals, thereby increasing the clinical severity and resulting in progressive respiratory failure [2, 8–10]. Severe neutropenia and lymphopenia often occur in patients with hematologic malignant neoplasms who receive a hematopoietic stem cell transplant (HSCT) [10, 11]. In HSCT recipients, severe LRT illness is associated with a mortality rate of up to 17% and occurs in up to 50% of HPIV infections [2, 3, 8, 12, 13]. The risk factors for LRT involvement and mortality include severe lymphopenia, respiratory coinfection, and receiving corticosteroid treatment at the time of HPIV infection [2, 3, 8, 11, 12, 14]. Immunosuppressive drugs that are required to kill leukemic cells in patients receiving chemotherapy also enhance LRT infection [15].

The dynamics of HPIV spread into the LRT and its subsequent clearance are unknown, largely because it is not possible to measure noninvasively the infection and host responses in the lungs of human subjects. With regard to animal models, cotton rats and hamsters are permissive but asymptomatic for HPIV infection, guinea pigs and ferrets are only semipermissive, and mice are poorly permissive [16]. A lack of disease in these models has been associated with limited HPIV replication, in part because interferon signaling is counteracted in a host-specific manner by human and animal PIVs [16]. Thus, it is impractical to study HPIV infection and the host response in small animal models, especially in the context of an immunocompromised host. As an alternative, Sendai virus (SeV) infection of mice has been used to study PIV spread and pathogenesis experimentally [17–20]. SeV is the murine counterpart of HPIV1. These viruses share 78% amino-acid sequence identity [21], elicit cross-protective immunity [22], have similar epidemiology and tropism [16, 18], and are thought to have similar transmission dynamics [20]. We previously engineered a non-attenuated luciferase reporter virus, designated rSeV-luc(M-F*), which we have used for noninvasive bioluminescence imaging of SeV infection in living, immunocompetent mice [19, 20, 23].

To our knowledge, there has been no previous noninvasive imaging study of respiratory viral infection in an immunocompromised host. We used the immunosuppressive drugs dexamethasone (Dexa) and cyclophosphamide (Cy) in doses and pulses that mimicked clinical drug schedules. Dexa is a potent steroid that is typically administered as a pulse therapy over several days to induce disease remission and maintain therapy when combined with other chemotherapeutic agents. Cy is commonly administered in combination with Dexa to treat hematologic malignant neoplasms. The goals of this study were to use bioluminescence imaging of SeV to investigate the mechanisms of viral spread into and clearance from the LRT in immunocompromised mice and to extend these findings by evaluating therapeutic interventions that compensate for immune components that are lacking.

## Results

### Immunosuppression after treatment with dexamethasone and/or cyclophosphamide

As shown in S1A Fig, we injected groups of BALB/c mice i.p. with Dexa (10 mg/kg daily for 10 days) and/or Cy (150 mg/kg on days 0 and 6). Control mice were injected i.p. with phosphate-buffered saline (PBS).

Treatment with Dexa and/or Cy induced weight loss of approximately 5% to 10% (*P* < 0.05) (S1B Fig). Treatment with Dexa alone reduced the peripheral lymphocyte count at 10 and 17 days after drug started (d.a.d.s.), whereas Cy administration (with or without Dexa) resulted in a maximal reduction in the lymphocyte count by 4 d.a.d.s. (*P* < 0.001) (S1C Fig). The lymphocyte count remained low after 17 days. Treatment with Dexa alone increased the peripheral neutrophil count at 4 and 10 d.a.d.s., whereas Cy (administered with or without Dexa) decreased the neutrophil count (*P* < 0.001) (S1D Fig). The neutrophil counts returned to pretreatment levels in all 3 treatment groups after 17 days. Overall, combining Cy with Dexa enhanced the immunosuppressive effects of the latter by accelerating the reduction in the lymphocyte count and causing neutropenia.

The spleen weight was reduced in drug-treated mice by 10 d.a.d.s. (*P* < 0.001) (S2A Fig) but increased thereafter. Dexa decreased the number of splenic lymphocytes (*P* < 0.01–0.001) (S2B Fig) but had no effect on splenic neutrophils at 13 and 16 d.a.d.s., whereas Cy treatment increased the splenic lymphocytes at 16 d.a.d.s. and neutrophils at 13 and 16 d.a.d.s. (S2B and S2C Fig). Dexa + Cy combination therapy resulted in a delay in the development of rebound splenomegaly relative to Cy alone and delayed the increase in spleen neutrophils and lymphocytes to day 16 (S2A–S2C Fig). Rebound increase in circulating neutrophils, splenic neutrophils and lymphocytes may be explained by increased colony-stimulating activity due to lack of feedback inhibition after cessation of chemotherapy [24].

### Dynamics of SeV infection in mice after immunosuppressive drug treatment

**Fig 1:**
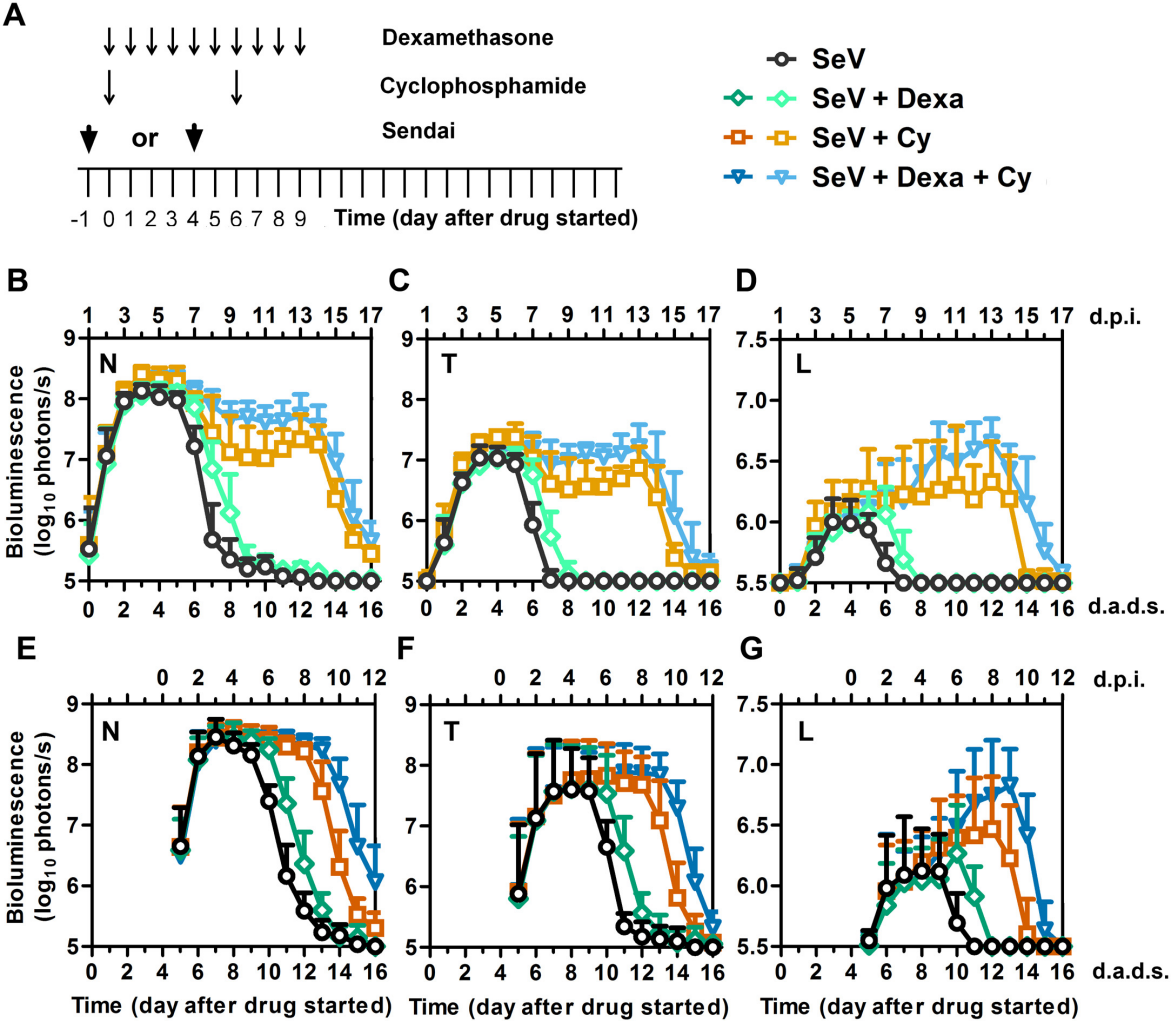
SeV infection measured by bioluminescence imaging in living mice after treatment with Dexa and/or Cy. (A) Drug treatment regimen and timing of infection. Arrows denote days on which drug injections were performed or 7,000 PFU Sendai virus (SeV) was intranasally inoculated in 5 μL PBS. Bioluminescence was measured after i.p. injection of 150 mg/kg D-luciferin and imaging with a Xenogen machine. (B–D) Bioluminescence of the nasal cavity (B), trachea (C), and lungs (D) in mice inoculated 1 day before starting drug treatment. Data shown are averages of 2 independent experiments with 10 mice at each time point. (E–G) Bioluminescence of the nasal cavity (E), trachea (F), and lungs (G) in mice inoculated 4 days after starting drug treatment. The data shown are averages of 3 independent experiments with 15 mice at each time point. For panels B-G, all groups were inoculated with SeV and symbols denote the following treatment groups: PBS (black circles), Dexa (green diamonds), Cy (orange squares), and Dexa + Cy (blue triangles). Lighter symbols in panels B-D correspond to groups inoculated with SeV 1 day before drug treatment, and darker symbols in panels E-G correspond to groups inoculated with SeV 4 days after drug treatment started. Error bars represent standard deviation. d.p.i., days postinfection; d.a.d.s., days after drug started.

We previously used noninvasive bioluminescence imaging to quantify the infection with a non-attenuated luciferase-expressing SeV reporter virus, designated rSeV-luc(M-F*), as a function of the mode of transmission (contact versus droplet), the inoculating dose and volume, the mouse strain, and prior exposure to SeV infection [19, 20, 23]. Here, we treated mice with Dexa and/or Cy and, either 1 day before or 4 days after starting the drug treatment, infected them with rSeV-luc(M-F*) in a 5-μL volume (Fig 1A). This inoculation method resulted in an initial infection dynamic similar to that observed after contact transmission in immunocompetent mice [23]. Dexa and/or Cy treatment had no apparent effect on the initial spread of the SeV infection (Fig 1B–1G), which in all groups began in the nasopharynx, spread 1 day later to the trachea and lungs, and reached a peak in the nasopharynx approximately 4 days postinfection (d.p.i.) (S3 Fig). Dexa and/or Cy treatment did not stimulate an acceleration of early phase infection in the URT and did not enhance early penetration of the infection into the LRT. This was not unexpected as Dexa causes apoptosis of lymphocytes [25, 26] and Cy is a cytotoxic alkylating agent that binds DNA to broadly suppress and delay B and T cell responses [27]. Consequently, immunosuppressive drug treatment caused marked delays in the clearance of the infection throughout the respiratory tract and enabled substantial increases in the extent of infection in the lungs. Regardless of whether the mice were infected with SeV one day before or 4 days after the start of Dexa treatment, the infection clearance was delayed by 1 day in the nasopharynx, trachea, and lungs (Fig 1B–1G and S4A Fig). Dexa treatment caused a small increase in the peak infection in the lungs but apparently caused no increase in infection in the nasopharynx or trachea. Dexa also caused a 1-day delay in clearance when administered in combination with Cy, as compared to treatment with Cy alone (Fig 1B–1G). Cy treatment resulted in prolonged infection, substantially increasing the level of infection in the lungs; this effect was enhanced when Dexa was combined with Cy (Fig 1D and 1G). In Cy- and Dexa + Cy-treated mice, the lung infection cleared by 8 and 9 days, respectively, after the final Cy treatment (14 and 15 d.a.d.s., respectively), regardless of whether the mice were infected 1 day before or 4 days after starting drug treatment (S4B and S4C Fig). As a 5-day delay in SeV inoculation had little effect on the timing of clearance, which coincided with the timing and end of Cy treatment, the data suggest that clearance may be associated with immunologic rebound as the effects of Cy subside. Consistent with this notion, continuing the pulses of Cy treatment at 5-day intervals resulted in prolonged infection (S4D Fig). Because the timing of clearance was independent of the timing of infection, we infected mice 4 days after the start of drug treatment in the remainder of this study.

### Bioluminescence correlates with viral load in immunosuppressed mice

**Fig 2:**
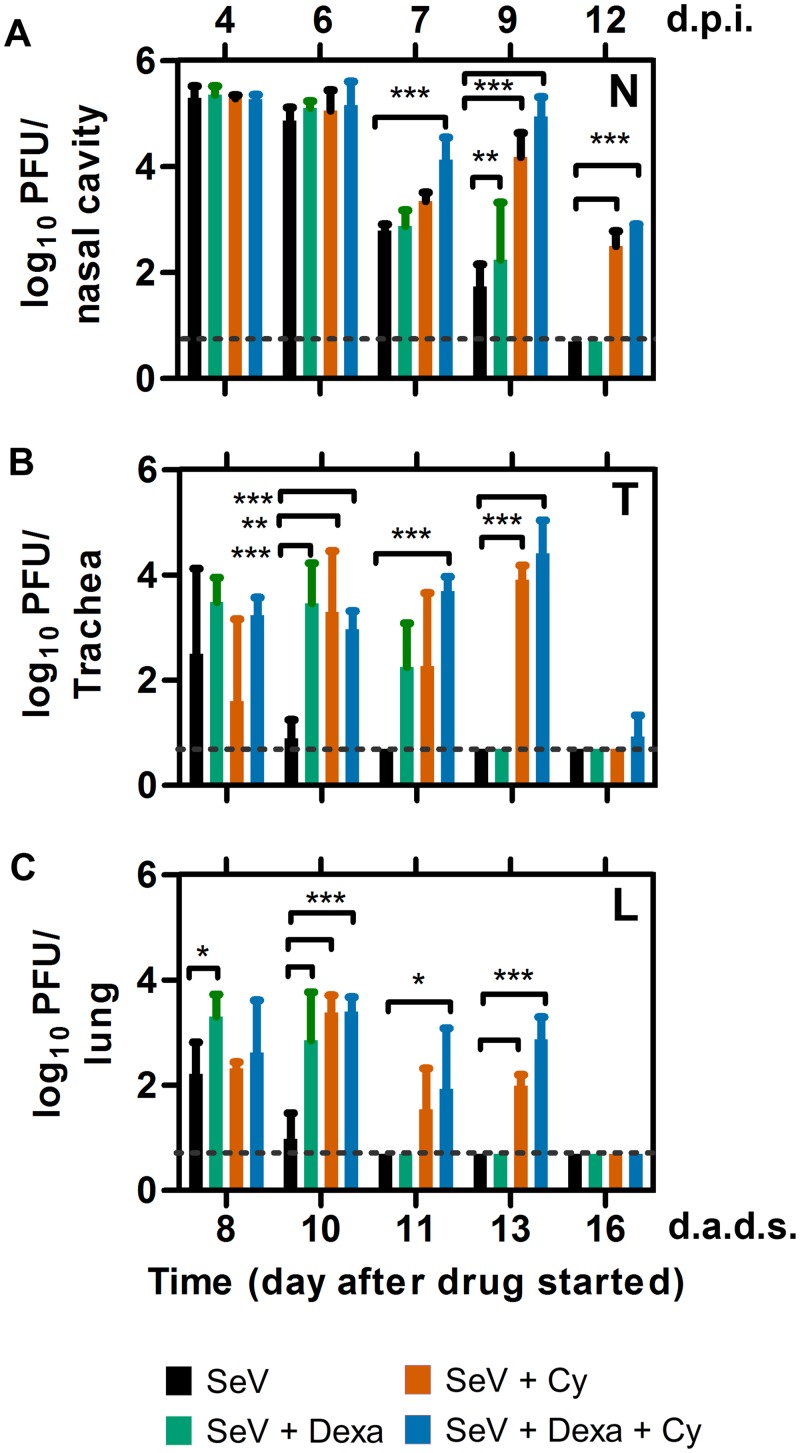
Viral load in the respiratory tracts of mice inoculated with SeV 4 days after treatment with Dexa and/or Cy. (A–C) Viral loads in the nasopharynx (A), trachea (B), and lungs (C). The data shown are averages of 3 animals at each time point. Bar colors for the groups are as follows: PBS (black), Dexa (green), Cy (orange), and Dexa + Cy (blue). Dashed lines represent the limit of detection. Error bars represent standard deviation. d.p.i., days postinfection; d.a.d.s., days after drug started. Statistics: 2-Way ANOVA; *P < 0.05, **P < 0.01, ***P < 0.001.

We previously showed that bioluminescence due to infection by rSeV-luc(M-F*) correlates with viral load in immunocompetent mice [19]. Here, we infected mice at 4 d.a.d.s. and collected respiratory tissues periodically to measure the viral loads. At 4 d.p.i., when the nasal bioluminescence peaked at a similar level for all groups (Fig 1E), the nasal viral load was similarly high and was equivalent independent of drug treatment (Fig 2A). Cy treatment resulted in delayed clearance of the nasal viral load, which had not yet been cleared at 12 d.p.i.; a similar timing was observed for the nasal bioluminescence. Viral loads in the trachea and lungs of PBS control mice started to clear at 6 d.p.i.; whereas, the start of clearance in the lungs of drug-treated mice was delayed until 7 d.p.i. (Fig 2B and 2C). Overall, the trends in the clearance of infection were consistent with respect to the viral load and bioluminescence data, which showed that partial nasal and full lung clearance had occurred 10 days after the final treatment with Cy (16 d.a.d.s).

### SeV-antigen staining in respiratory tissues correlates inversely with T cell infiltration

**Fig 3:**
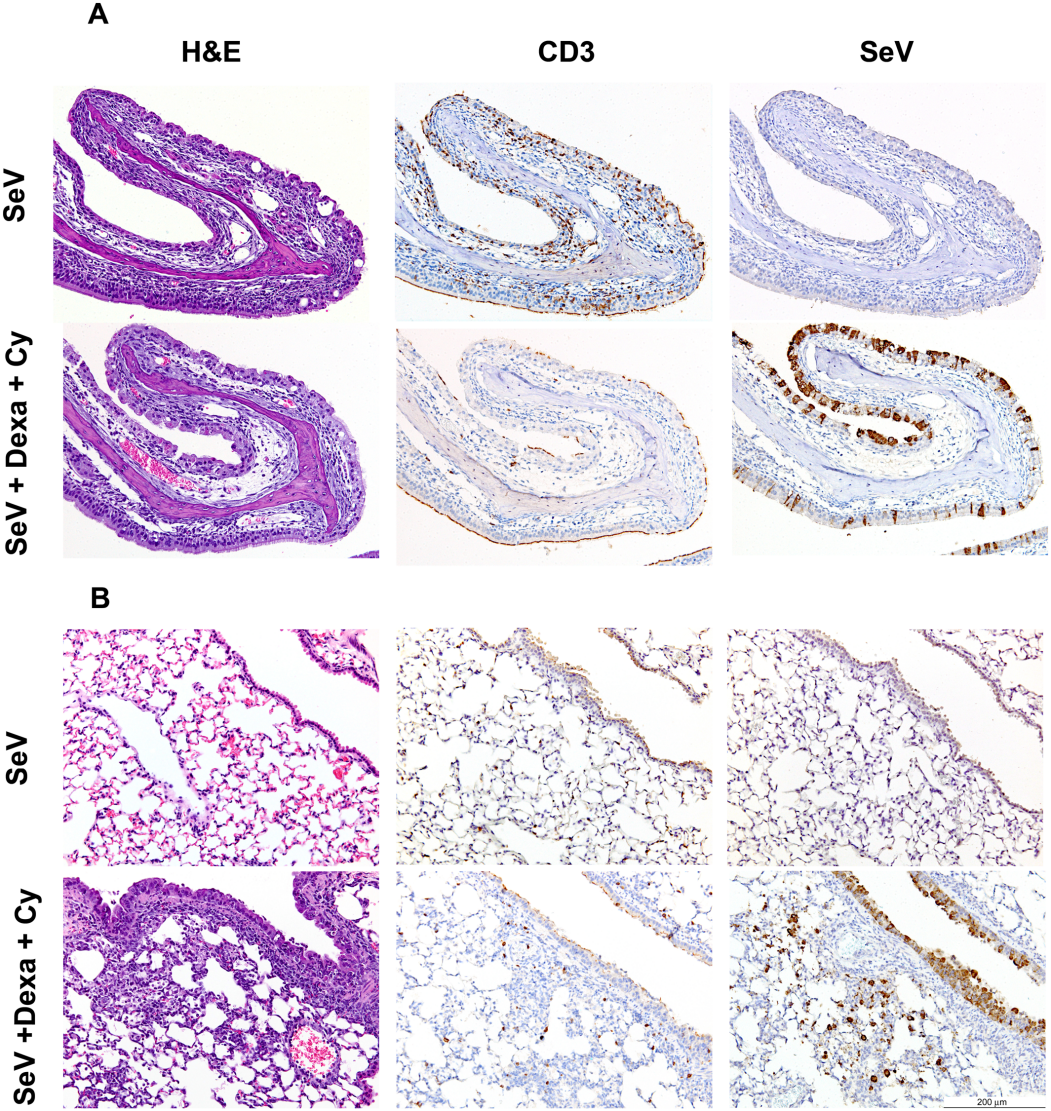
Histopathologic changes 13 d.a.d.s. (9 d.p.i.) in the respiratory tracts of mice inoculated with SeV 4 days after treatment with Dexa and /or Cy. Sections of the nasal cavity (A) and lungs (B) were stained with hematoxylin and eosin (H&E) (left panels), with a mAb to CD3 to show T-cell infiltration (middle panels), or with a mAb to SeV (right panels). Sections from Dexa + Cy-treated mice (bottom panels) were compared to sections from untreated controls (upper panels). The data are representative of the 4 different animals in each group.

To compare local respiratory infection and cellular infiltration data to the noninvasive bioluminescence data, we infected the mice that had been treated Dexa and Cy (or PBS) at 4 d.a.d.s. and collected respiratory tissues for histopathologic analysis. At 6 d.p.i. in non-drug-treated mice, the nasal bioluminescence started to clear, SeV-infected cells and infiltrating T cells were observed, and the nasal epithelium showed marked disruption with scattered apoptotic epithelial cells (Fig 1E and S5 Fig). In the Dexa + Cy-treated group at 6 d.p.i., the nasal bioluminescence continued at near peak levels, and numerous SeV-positive cells were observed; however, there was minimal disruption of the nasal epithelium and minimal inflammation, with no to minimal foci of infiltrating T cells and macrophages. At 9 d.p.i. in non-drug-treated mice, the nasal bioluminescence had cleared, and minimal viral antigen was detected in the nasal cavity (Figs 1E and 3A). Similar to 6 d.p.i., the nasal epithelium remained disrupted, and there was a diffuse increase in T cells. In the Dexa + Cy-treated group at 9 d.p.i., the nasal bioluminescence continued at near peak levels, SeV-positive cells were abundant in the nasal cavity, and there was mild inflammation and rare T-cell infiltration (Figs 1E and 3A). At 12 d.p.i., the nasal bioluminescence was largely cleared, SeV antigen was only detected in the olfactory neuroepithelium, inflammation was minimal, and numerous T cells were scattered throughout the nasal submucosa (S5B Fig).

With respect to the LRT, at 9 d.p.i. in non-drug-treated mice, the lung bioluminescence was cleared, no SeV antigen was observed in the lungs, and T cells were scattered in the submucosa of the lung airways (Figs 1G and 3B). In the Dexa + Cy-treated group at 9 d.p.i., the lung bioluminescence was at its peak, SeV-infected cells were abundant in the airways and scattered in a few terminal bronchioles but were not seen in the alveoli, and a minimal number of T cells were scattered in the pulmonary submucosa. In both the untreated and drug-treated groups, no neutrophilic infiltration was observed in the nasal cavity, trachea, or lungs between 6 and 12 d.p.i. Overall, the bioluminescence and SeV antigen staining results were consistent with each other and were inversely correlated with inflammation and T-cell infiltration.

### Immunosuppression and the relationship between immune reconstitution and clearance of infection

**Fig 4:**
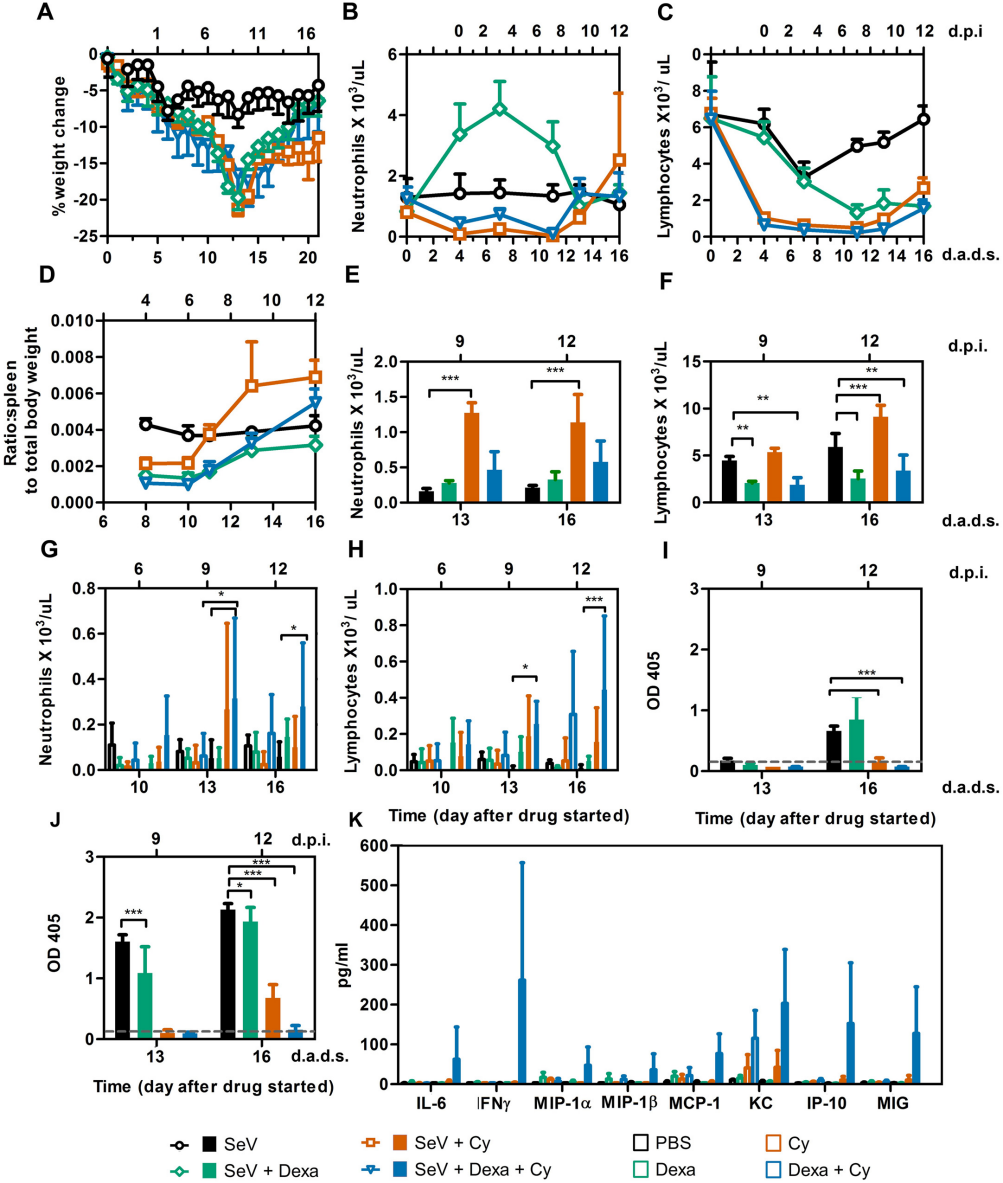
Immunological responses in immunosuppressed mice inoculated with SeV at 4 d.a.d.s. (A) Percent change in starting weight. (B) Peripheral blood neutrophil counts. (C) Peripheral blood lymphocyte counts. (D) Ratio of spleen weight to total body weight. For panels A-D, groups are shown as follows: PBS (black circles), Dexa (green diamonds), Cy (orange squares), and Dexa + Cy (blue triangles). (E) Splenic neutrophil counts. (F) Splenic lymphocyte counts. (G) BALF neutrophil counts. (H) BALF lymphocyte counts. (I and J) SeV-specific IgG levels in the BALF (I) and serum (J) of mice infected with SeV 4 days after treatment with Dexa and/or Cy. (K) Chemokines/cytokines in the BALF of SeV-infected mice at 12 d.p.i. (16 d.a.d.s). For panels E-K, bars are colored as follows: PBS (black), Dexa (green), Cy (orange), and Dexa + Cy (blue). The data shown are a representative of 2 independent experiments with 3 to 5 mice in each group. Error bars represent standard deviation. d.p.i., days postinfection; d.a.d.s., days after drug started. Statistics: 2-way ANOVA; *P < 0.05, **P < 0.01, ***P < 0.001.

As the timing of clearance coincided with the time after Cy treatment stopped and not the timing of SeV inoculation, we hypothesized that clearance is driven by immune reconstitution, and we wished to investigate its mechanism. We inoculated groups of mice intranasally, monitored them for clinical symptoms, collected their peripheral blood periodically, and euthanized some of the animals to collect tissues for analysis. In the non-drug-treated groups, uninfected mice showed no weight change (S1B Fig), whereas SeV-infected mice exhibited a maximum weight loss of approximately 10% (Fig 4A). Uninfected, immunosuppressed mice lost approximately 5% to 10% of their body weight, whereas those that were immunosuppressed and infected lost approximately 20% of their body weight. In immunocompetent SeV-infected mice, the number of peripheral neutrophils was unchanged, whereas peripheral lymphocytes were reduced 2-fold, with a nadir at 3 d.p.i. (7 d.a.d.s.), and recovered over the next 9 days (Fig 4B and 4C). Dexa treatment resulted in a 3-fold increase in peripheral neutrophils, which peaked at 3 d.p.i. (7 d.a.d.s., *P* < 0.001) and an approximately 4-fold decrease in peripheral lymphocytes between 7 and 12 d.p.i. (11–16 d.a.d.s., *P* < 0.001). Therefore, when the clearance of infection began on 11 d.a.d.s. in Dexa-treated mice (Fig 1E–1G), the mice had an increased number of neutrophils but were suffering from lymphopenia (Fig 4B and 4C). Cy treatment (with or without Dexa) resulted in lymphopenia (*P* < 0.001) and neutropenia (*P* < 0.01) by 4 d.a.d.s. (i.e., the day of inoculation). The lymphopenia continued throughout the experiment, whereas the peripheral neutrophil counts were restored at 13 d.a.d.s. (Fig 4B and 4C), coinciding with the timing of clearance (Fig 1E–1G). Overall, clearance coincided with the recovery of peripheral neutrophils, which occurred simultaneously with a rebound in the spleen weight and splenic neutrophil count in Cy-treated mice (*P* < 0.001) (Fig 4D–4F), as described previously [28]. In this study, we also noted that adding Dexa to the Cy treatment dampened the rebound of splenic lymphocytes (*P* < 0.01) (Fig 4F) and delayed the rebound of splenic granulocytes (Fig 4E), whose levels were similar to uninfected animals.

Next, we compared the infiltration of immune cells into bronchoalveolar lavage fluid (BALF) and LRT clearance. Between 13 and 16 d.a.d.s., we observed significant increases in the lymphocyte and neutrophil counts in BALF in only Dexa + Cy-treated mice, as compared to immunocompetent, infected control animals (*P* < 0.05–0.001) (Fig 4G and 4H). During this time interval, the lung infection was cleared from Dexa + Cy-treated mice (Fig 1G) as the immune cell cellularity increased in the lungs (Fig 3B). However, by 16 d.a.d.s., no antibody responses were yet detectable in the BALF or peripheral sera (Fig 4I and 4J). Dexa-treated mice generated antibody responses that were robust, albeit significantly weaker than those in immunocompetent control animals, and most likely contributed to clearance, which was complete by 13 d.a.d.s. (*P* < 0.05–0.001) (Fig 1E–1G).

We measured the levels of 32 pro- and anti-inflammatory cytokines and chemokines in the BALF and sera on 10, 13, and 16 d.a.d.s. For SeV-infected mice, each of the 32 inflammatory mediators was not significantly different (*P* > 0.05) between groups either untreated or treated with Dexa. In Cy-treated and SeV-infected mice at 13 d.a.d.s., of the 32 inflammatory mediators tested only IP-10 was significantly increased (*P* < 0.01) compared to controls (infected/non-drug-treated and uninfected/drug-treated). In Dexa + Cy-treated mice, lung infection was more prominent and was cleared between 13 and 16 d.a.d.s. In this group, significant increases in expression of 8/32 inflammatory mediators were observed at 16 d.a.d.s., including IL-6, IFNγ, MIP-1α, MIP-1β, MCP-1, KC, IP-10, and MIG (*P* < 0.01–0.001) (Fig 4K). Inflammatory mediators not differing significantly from control groups were omitted from Fig 4K for simplicity. Because of the mild infection in the lungs of non-drug-treated/SeV-infected mice, an attenuated inflammatory response in BALF resulted in only IL-13 and IL-15 (out of the 32 tested) being significantly increased compared to PBS-inoculated control mice (*P* < 0.01). Overall, the magnitude of cytokine and chemokine expression was proportional to the level of pulmonary infection, and the timing of the expression of inflammatory mediators coincided with the increases in cellular infiltration and inflammation noted by histopathologic analysis.

### Neutrophils are neither necessary nor sufficient for clearance in an immunocompromised host

**Fig 5:**
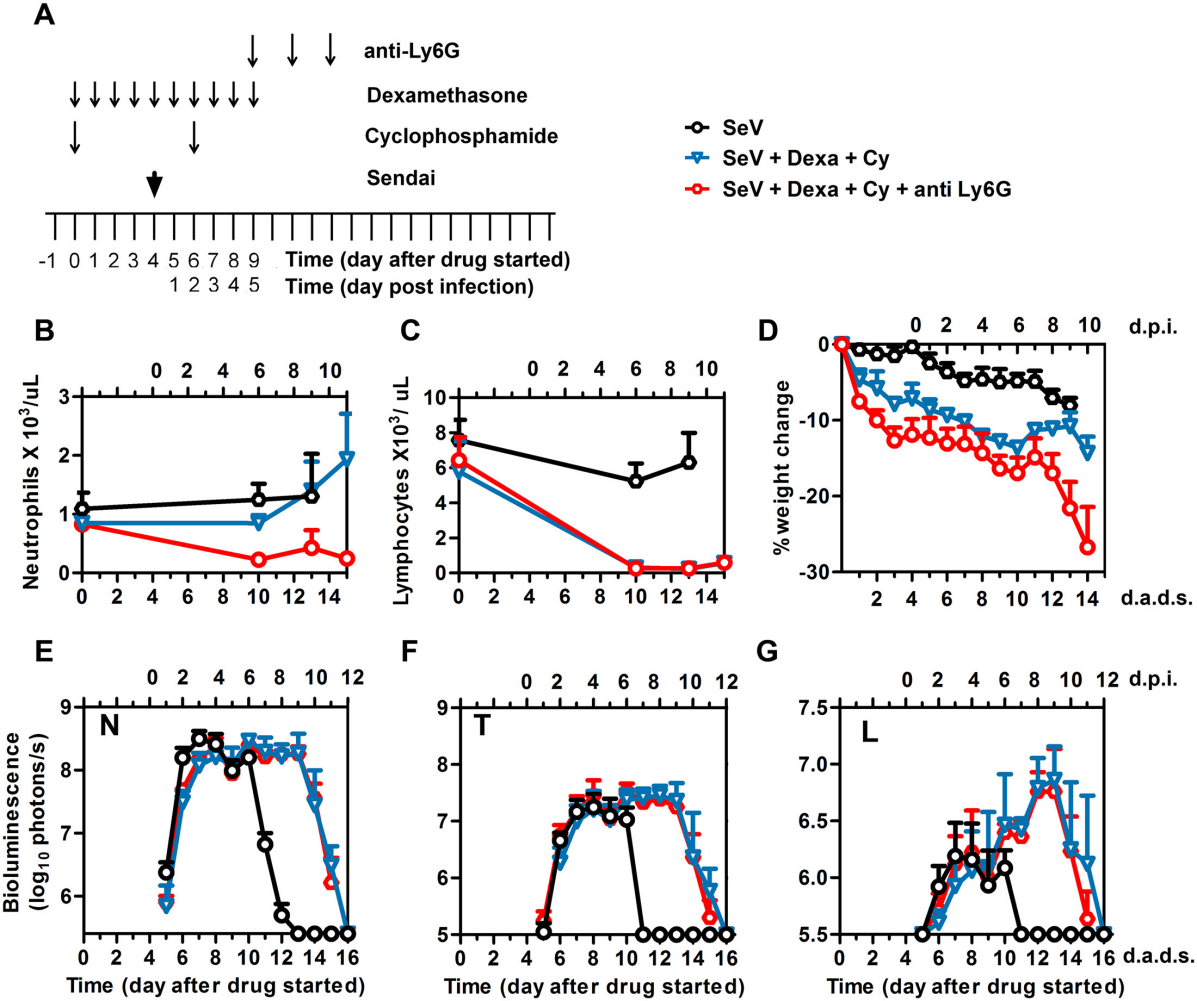
Effect of anti-neutrophil antibody Ly6G on viral clearance. (A) Drug treatment regimen with anti-neutrophil antibody (anti-Ly6G). Arrows denote days on which anti-Ly6G was i.p. injected, drug injections were performed, and 7,000 PFU Sendai virus (SeV) was intranasally inoculated in 5 μL PBS. (B) Peripheral blood neutrophil counts. (C) Peripheral blood lymphocyte counts. (D) Percent change in starting weight. (E-G) Bioluminescence in the nasopharynx (E), trachea (F), and lungs (G). Groups include PBS (black circles), Dexa + Cy (blue triangles), and Dexa + Cy + anti-Ly6G (red circles). The data shown are averages of 5 mice from each group. In all graphs, error bars represent the standard deviation. d.p.i., days postinfection; d.a.d.s., days after drug started.

Respiratory viral infection increases expression of cytokines within the respiratory tract, which helps trigger infiltration of innate immune cells including neutrophils. This plays a critical role in the initiation of the adaptive immunity [29]. The role of neutrophils in respiratory viral infection has been poorly defined. An earlier study negated a role for neutrophils in clearing influenza A virus infection [30], while later studies have suggested a role of neutrophils in guiding and maintaining CD8+ T cells in the airways during an infection [31, 32]. A role for neutrophils in counteracting PIV in an immunocompromised host was unknown. Based on the observed coincidence of viral clearance with neutrophil rebound in blood, spleen, and BALF, we hypothesized that neutrophils provide a therapeutic benefit in the immunocompromised host. To determine if neutrophil rebound was necessary for clearance in Dexa + Cy-treated mice, we treated mice with these drugs, infected them with rSeV-luc(M-F*) at 4 d.a.d.s., and depleted their neutrophils with 0.5 mg of α-Ly6G antibody administered i.p. at 5, 7, and 9 d.p.i. (Fig 5A). The resulting peripheral blood neutrophil counts were significantly lower than in mice not treated with the antibody (*P* < 0.001) (Fig 5B), and the lymphocyte counts remained low (Fig 5C). We observed no difference in viral clearance in the nasopharynx, trachea, or lungs with or without neutrophil depletion (Fig 5E–5G), showing neutrophils are not necessary for clearance in the immunosuppressed host. α-Ly6G has been reported to cause morbidity [31–33]. In our studies α-Ly6G-treated mice lost significantly more weight relative to control mice (*P* < 0.01, Fig 5D) and 100% had to be euthanized for excess morbidity after 14 d.a.d.s. while all of the control mice survived.

**Fig 6:**
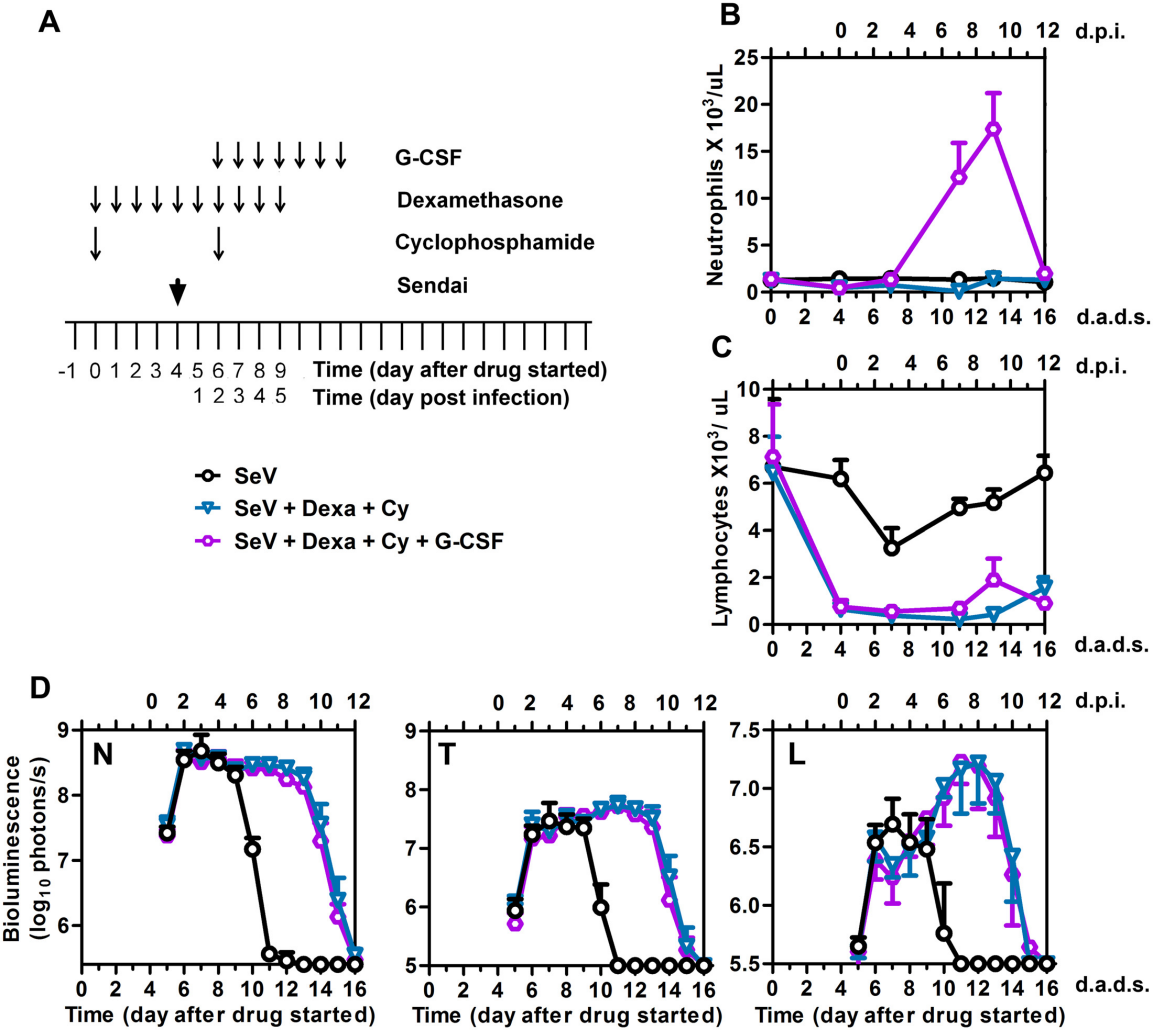
Effect of granulocyte-colony stimulating factor (G-CSF) on viral clearance. (A) Drug treatment regimen with G-CSF. Arrows denote days on which G-CSF was i.p. injected, drug injections were performed, and 7,000 PFU Sendai virus (SeV) was intranasally inoculated in 5 μL PBS. (B) Peripheral blood neutrophil counts. (C) Peripheral blood lymphocyte counts. (D) Bioluminescence in the nasopharynx (N), trachea (T), and lungs (L). Groups include PBS (black circles), Dexa + Cy (blue triangles), and Dexa + Cy + G-CSF (purple circles). The data shown are a representative of 2 independent experiments with 5 mice per group. In all graphs, error bars represent the standard deviation. d.p.i., days postinfection; d.a.d.s., days after drug started.

To determine if neutrophil stimulation was sufficient to promote SeV clearance in the immunocompromised host, we treated mice with Dexa and Cy, infected at 4 d.a.d.s., and stimulated the neutrophil counts by administering G-CSF i.p. for 7 days starting at 2 d.p.i. (Fig 6A). G-CSF is a growth factor that is able to enhance the growth of mainly the neutrophilic colonies of granulocytes, enhance their binding to chemotactic factors, and shorten the periods of neutropenia after chemotherapy [34, 35]. Currently, G-CSF is used clinically to accelerate the recovery of the peripheral neutrophils and to reduce the rates of infection after intensive chemotherapy treatment of hematologic malignancies and HSCT. A deficiency of G-CSF during SeV infection has been reported to decrease survival [36]; however, to our knowledge the benefit of G-CSF administration to prevent or treat lung infection has not been tested. G-CSF treatment increased peripheral neutrophil counts (*P* < 0.001) (Fig 6B), and lymphocyte counts remained low (Fig 6C). We observed no difference in viral clearance from the nasopharynx, trachea, or lungs in the presence or absence of G-CSF (Fig 6D), showing that neutrophils are not sufficient to enhance viral clearance in the immunocompromised host.

We also investigated the use GM-CSF as a possible alternative therapeutic intervention to G-CSF. GM-CSF is a pleiotropic cytokine that can stimulate cells of the myeloid lineage, although less potently than G-CSF [37], and has been shown to be protective against influenza A virus infection [38]. In the context of SeV infection, GM-CSF had no apparent effect on viral clearance in the nasopharynx, trachea, and lungs. This was the case whether GM-CSF was administered either intranasally at a dose of 100 ng/ mouse/ day for 7 days starting at 6 d.a.d.s. (S6A–S6C Fig) or i.p. at a dose of 200 ng/ mouse/ day for 7 days starting at 6 d.a.d.s. (within the range of the dose used clinically [39]) (S6H–S6J Fig). Intranasal administration of GM-CSF resulted in less inflammatory cell recruitment (S6D–S6F Fig) and less inflammatory chemokine expression of IP-10 in the BAL at 14 d.a.d.s. (S6G Fig). This suggests GM-CSF plays a role in controlling the inflammatory response, especially after repeated intranasal administration of an infected animal.

### Viral clearance coincides with a rebound in peripheral natural killer (NK) cells and is delayed by NK cell depletion

**Fig 7:**
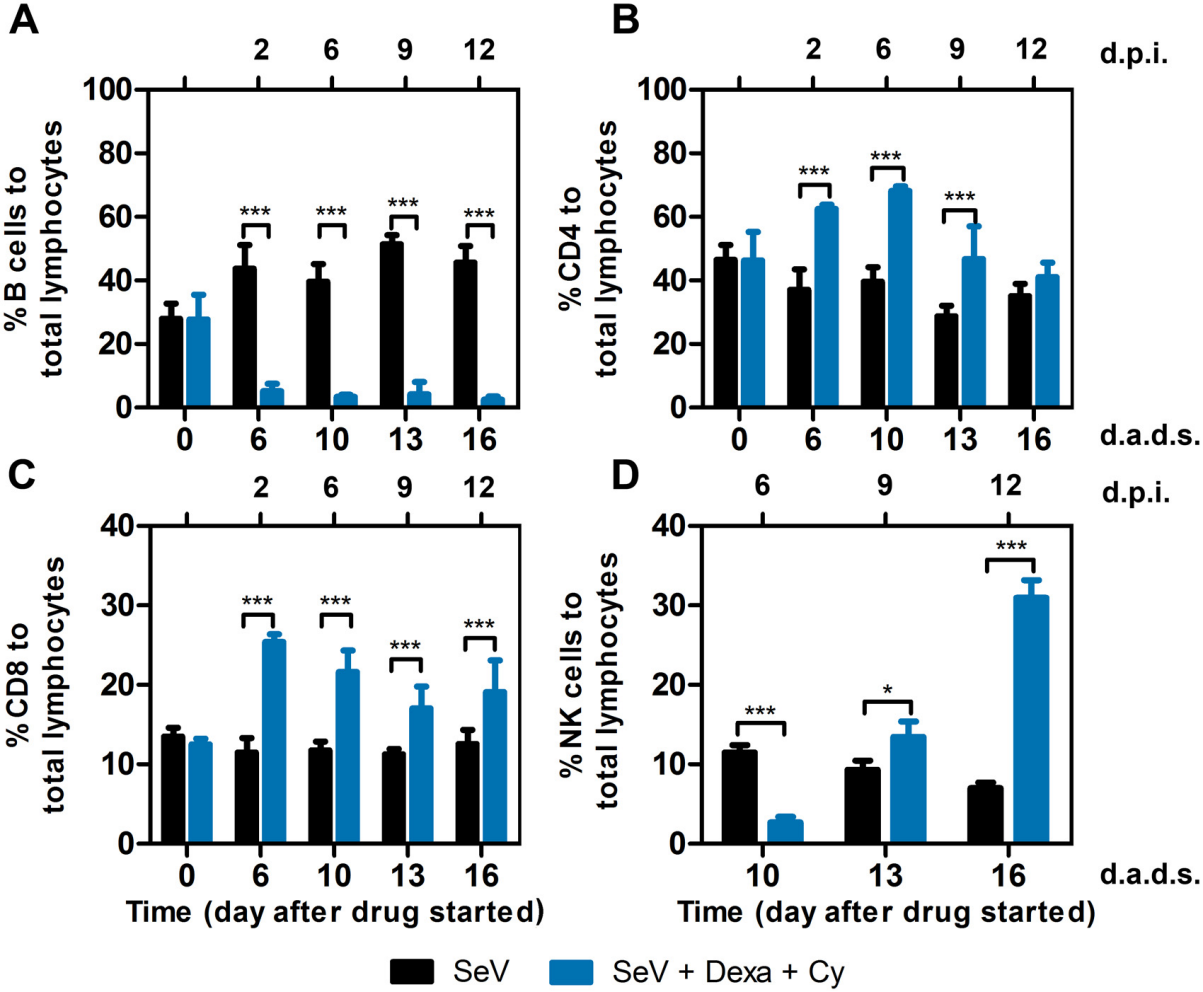
Effect of drug treatment on peripheral lymphocyte populations. (A) B cell, (B) CD4+ T cell, (C) CD8+ T cell, and (D) NK cell percentages were determined for peripheral blood collected at the indicated time points. Dexa and Cy injections were performed as described previously, and 7,000 PFU SeV was intranasally inoculated in 5 μL PBS at 4 d.a.d.s. Groups include PBS (black bars) and Dexa + Cy (blue bars). The data shown are averages of 5 mice per group. In all graphs, error bars represent the standard deviation. d.p.i., days postinfection; d.a.d.s., days after drug started. * *P* < 0.05, *** *P* < 0.001.

**Fig 8:**
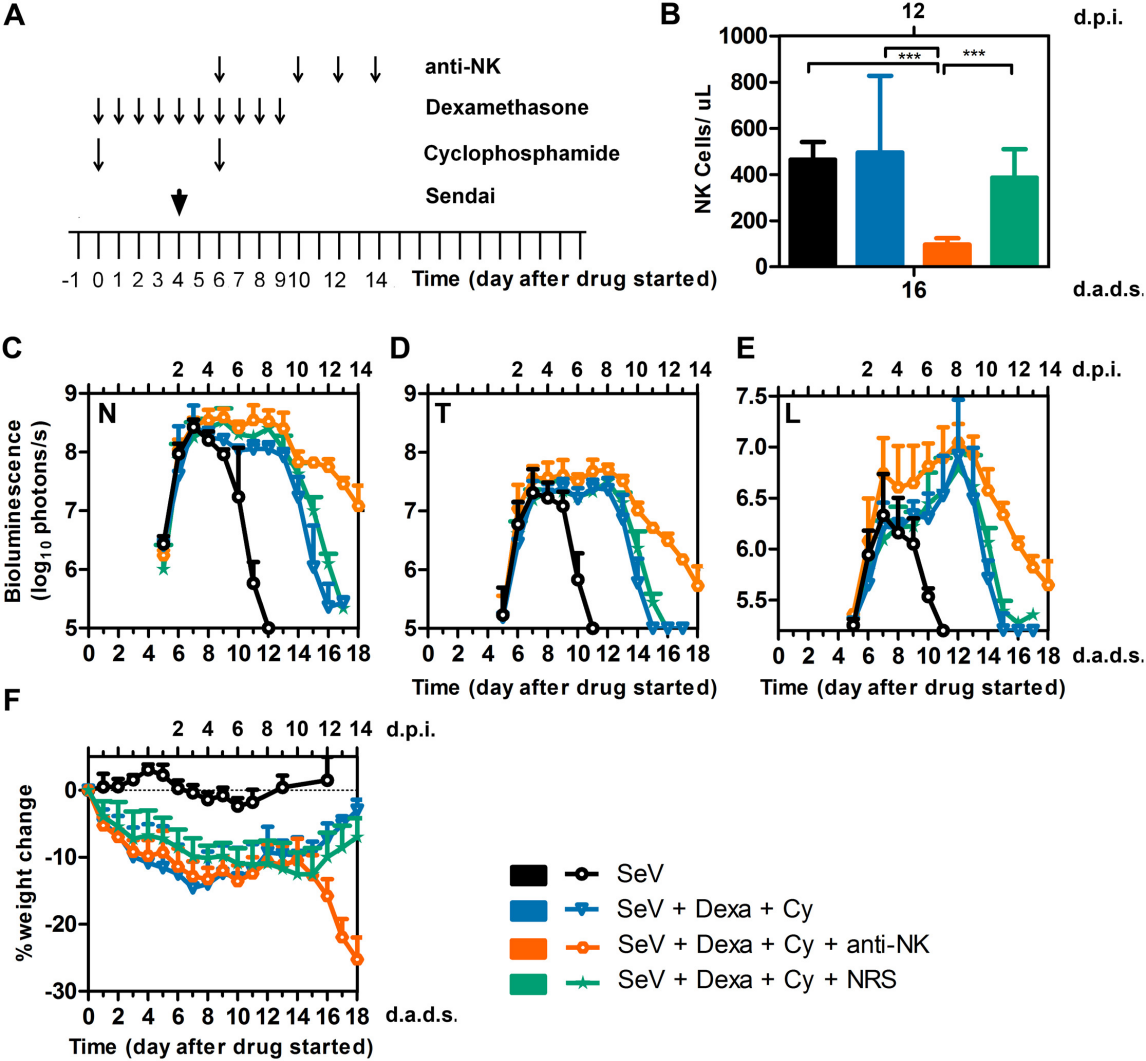
Effect of anti-NK (anti-asialo GM1) antibody on viral clearance. (A) Drug treatment regimen with anti-NK antibody. Arrows denote days on which anti-NK or normal rabbit serum (NRS) were i.p. injected, drug injections were performed, and 7,000 PFU SeV was intranasally inoculated in 5 μL PBS. (B) Peripheral NK cell absolute numbers at 16 d.a.d.s. (C-E) Bioluminescence in the nasopharynx (C), trachea (D), and lungs (E). (F) Percent change in starting weight. Groups include PBS (black bars and circles), Dexa + Cy (light blue bars and triangles), Dexa + Cy + anti-NK (orange bars and octagons), and Dexa + Cy + NRS (green bars and stars). The data shown are averages of 5 mice per group. In all graphs, error bars represent the standard deviation. d.p.i., days postinfection; d.a.d.s., days after drug started. *** *P* < 0.001.

To analyze the effect of drug treatments on different populations of lymphocytes and correlate the initiation of viral clearance with the reconstitution of a specific lymphocyte population, we treated mice with Dexa + Cy, infected at 4 d.a.d.s., collected blood at various time points, and examined lymphocyte populations by flow cytometry. Drug treatment significantly reduced the percentage of B-lymphocytes (Fig 7A, *P* < 0.001), consistent with substantial reductions in serum and local antibody responses (Fig 4I and 4J). Viral clearance occurred during B-cell depletion. Drug treatment significantly increased the percentages of both CD4^+^ T cells at 6, 10, and 13 d.a.d.s. and CD8^+^ T cells at 6, 10, 13, and 16 d.a.d.s. (Fig 7B and 7C, *p* < 0.001). Drug treatment resulted in an initial reduction in NK-cell percentages (10 d.a.d.s., *p* < 0.001) and a later increase (days 13, *p* < 0.05 and 16, *p* < 0.001, Fig 7D). The timing of the increase in the NK cell population after the discontinuation of Dexa + Cy correlated with viral clearance. To determine if NK rebound promoted viral clearance, we treated mice with Dexa + Cy, infected at 4 d.a.d.s., and treated with the anti-asialo GM1 antibody, which completely abolishes NK cell activity in BALB/c mice [40, 41], at days 6, 10, 12, and 14 d.a.d.s. (Fig 8A). NK cells were reduced approximately 5 fold relative to both non-drug treated and Dexa + Cy treated groups in the peripheral blood (Fig 8B, *P* < 0.001). NK-cell depletion resulted in delayed clearance of nasal, tracheal, and lung infection (*P* < 0.05–0.001) (Fig 8C–8E). NK-depleted mice started to rapidly lose body weight after 15 d.a.d.s. (*P* < 0.001), suggesting NK cells may help regulate the inflammatory response during viral clearance (Fig 8F). Overall, the data demonstrates a role for NK cells in efficient viral clearance.

### T-lymphocytes are necessary for viral clearance

**Fig 9:**
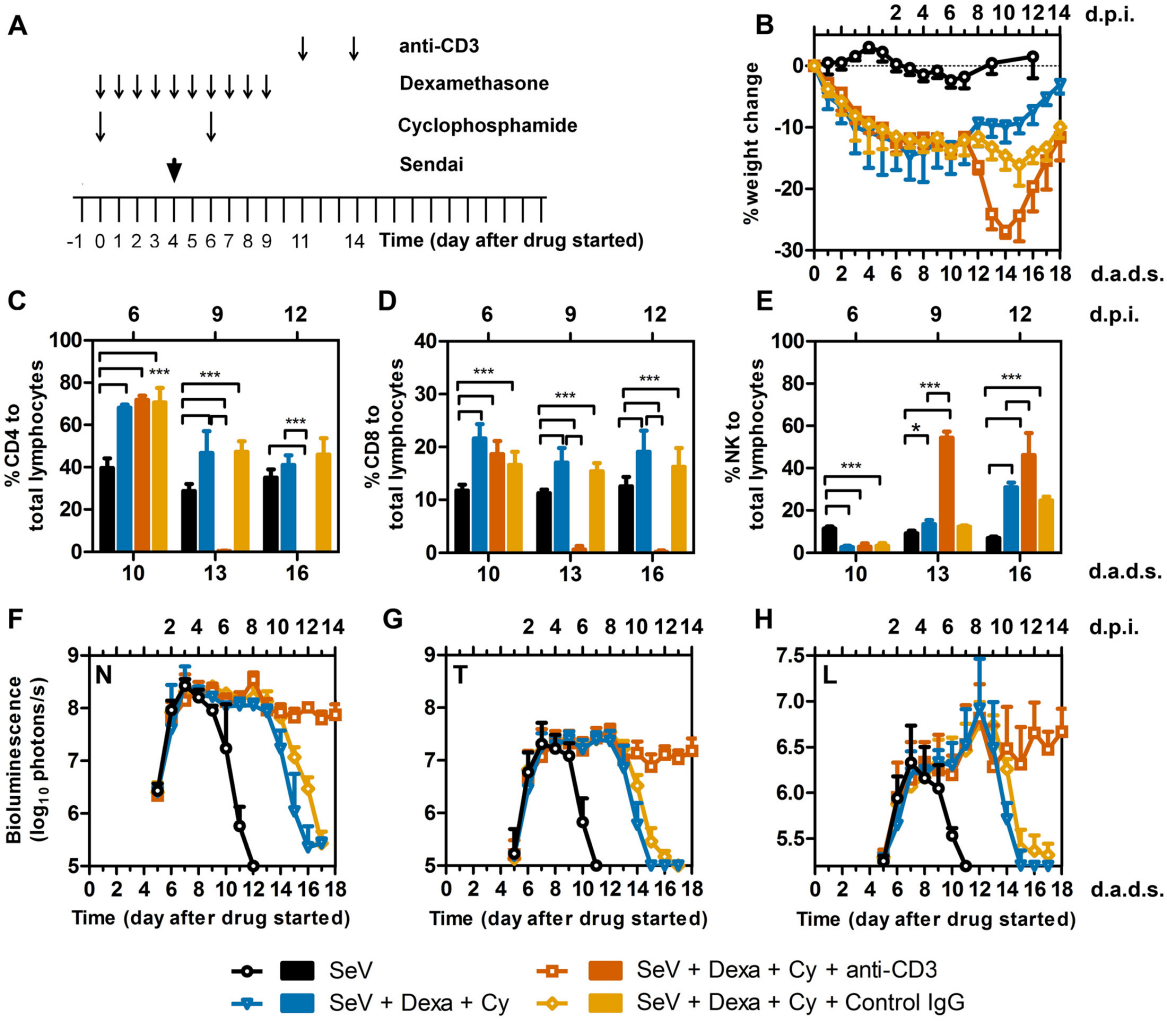
Effect of anti-CD3 (anti-T cell) antibody on viral clearance. (A) Drug treatment regimen with anti-CD3 antibody. Arrows denote days on which anti-CD3 or isotype control IgG were i.p. injected, drug injections were performed, and 7,000 PFU SeV was intranasally inoculated in 5 μL PBS. (B) Percent change in starting weight. (C-E) Percentages of (C) CD4+ T cells (D) CD8+ T cells, and (E) NK cells in peripheral blood. (F-H) Bioluminescence in the nasopharynx (F), trachea (G), and lungs (H). Groups include PBS (black bars and circles), Dexa + Cy (light blue bars and triangles), Dexa + Cy + anti-CD3 (burnt orange bars and rectangles), control IgG (orange bars and diamonds). The data shown are averages of 5 mice per group. In all graphs, error bars represent the standard deviation. d.p.i., days postinfection; d.a.d.s., days after drug started. * p < 0.05, *** p < 0.001.

To analyze the contribution of T lymphocytes to viral clearance, we treated mice with Dexa + Cy, infected at 4 d.a.d.s., and treated with two 20-μg doses of anti-CD3 antibody, administered i.p. at 11 and 14 d.a.d.s. (Fig 9A). After the first dose of the antibody, the mice progressively lost weight (starting at 12 d.a.d.s.), but rapidly recovered weight after 14 d.a.d.s. (Fig 9B). This treatment regimen caused a significant reduction in the T cell population, and led to an increase in the NK cell population (Fig 9C–9E). Mice depleted of T cells were not able to clear viral infection and had a continuously high signal that was significantly higher than the Dexa + Cy group after 13 d.a.d.s. (Fig 9F–9H, *P* < 0.001). Overall, T-lymphocytes were shown to be critical for viral clearance as T-cell-depleted mice were unable to clear the infection.

### Therapeutic antibodies protect from high levels of lung infection in an immunocompromised host

**Fig 10:**
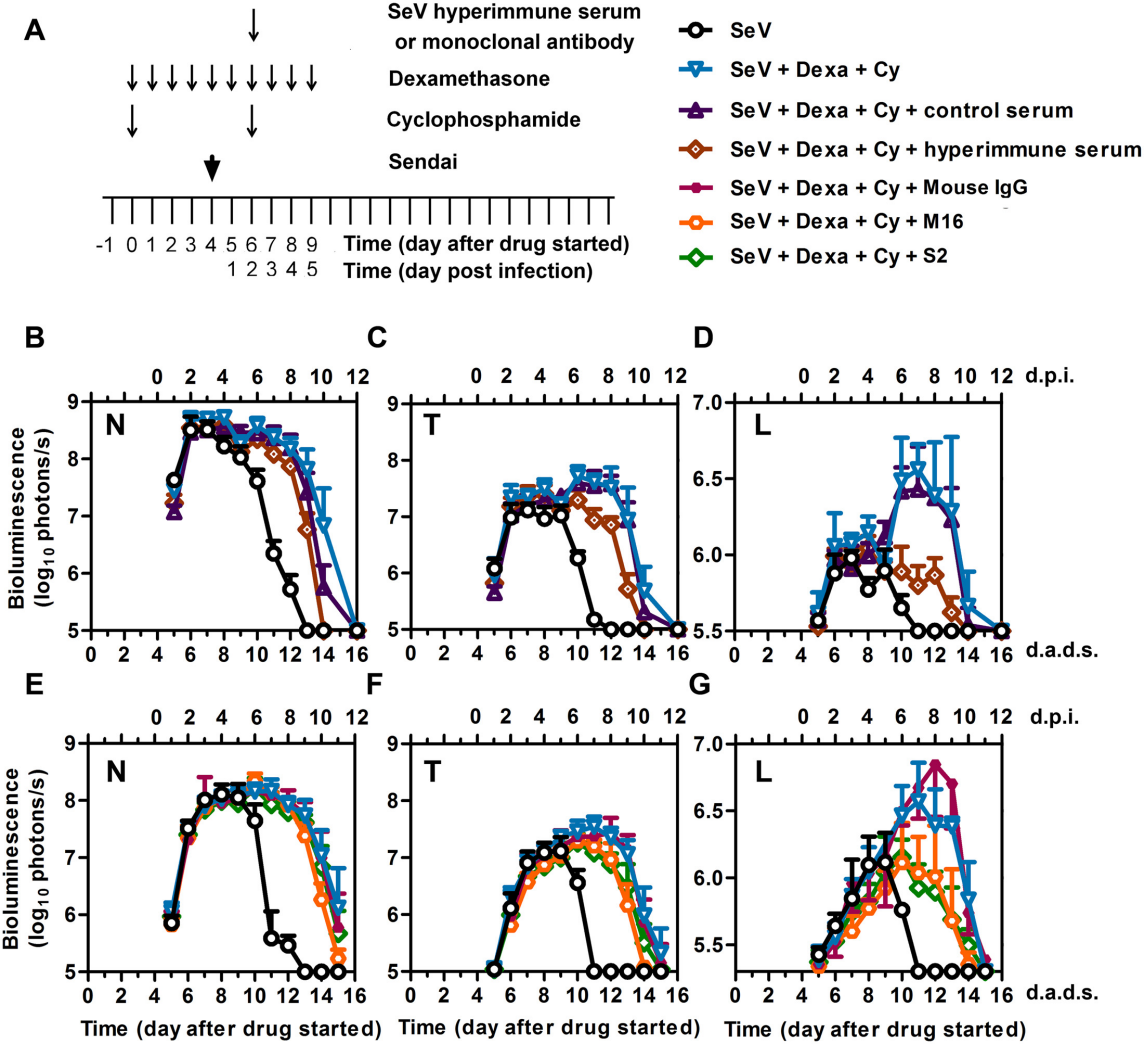
Effects of SeV-specific hyperimmune pooled serum and monoclonal antibodies on controlling SeV infection in immunosuppressed mice. (A) Drug treatment regimen with SeV-specific hyperimmune serum or mAbs. Arrows denote days on which serum or monoclonal antibodies were i.p. injected, drug injections were performed, and 7,000 PFU Sendai virus (SeV) was intranasally inoculated in 5 μL PBS. (B–D) Bioluminescence in the nasopharynx (B), trachea (C), and lungs (D) after infection with SeV and the following conditions: no immunosuppression and no hyperimmune serum (black circles), Dexa + Cy without hyperimmune serum (blue triangles), Dexa + Cy with hyperimmune serum (brown diamonds), and Dexa + Cy with nonspecific control serum (dark purple triangles). (E-G) Bioluminescence in the nasopharynx (E), trachea (F), and lungs (G) after infection with SeV and the following conditions: no immunosuppression and no monoclonal antibodies (black circles), Dexa + Cy without monoclonal antibodies (blue triangles), Dexa + Cy + M16 [anti-F protein monoclonal antibody] (orange circles), Dexa + Cy + S2 [anti-HN protein monoclonal antibody] (green diamonds), and Dexa + Cy + isotype control mouse IgG (small purple squares). The data shown are averages of 5 mice per group. In all graphs, error bars represent the standard deviation. d.p.i., days postinfection; d.a.d.s., days after drug started.

To determine if antibodies are sufficient to limit infection when administered therapeutically, we treated mice with Dexa and Cy, infected at 4 d.a.d.s., and treated at 2 d.p.i. (6 d.a.d.s.) with i.p.-administered hyperimmune serum pooled from SeV-immunized mice (Fig 10A). Hyperimmune serum markedly reduced the bioluminescence in the lungs and modestly reduced that in the nasopharynx and trachea, accelerating the viral clearance by 2 days, as compared to that in controls (*P* < 0.001) (Fig 10B–10D). Using an identical schedule, we also treated mice with the mAb M16 (α-SeV F protein), the mAb S2 (α-SeV HN protein), or nonspecific mouse IgG administered i.p. at 2 d.p.i. (6 d.a.d.s.). Both SeV-specific mAbs caused substantial reductions in the lung bioluminescence and more modest reductions in the trachea and nasopharynx, accelerating the clearance by 1 day, as compared to that in controls (*P* < 0.001) (Fig 10E–10G).

### Protective immunity is elicited after recovery from immunosuppression and SeV infection

**Fig 11:**
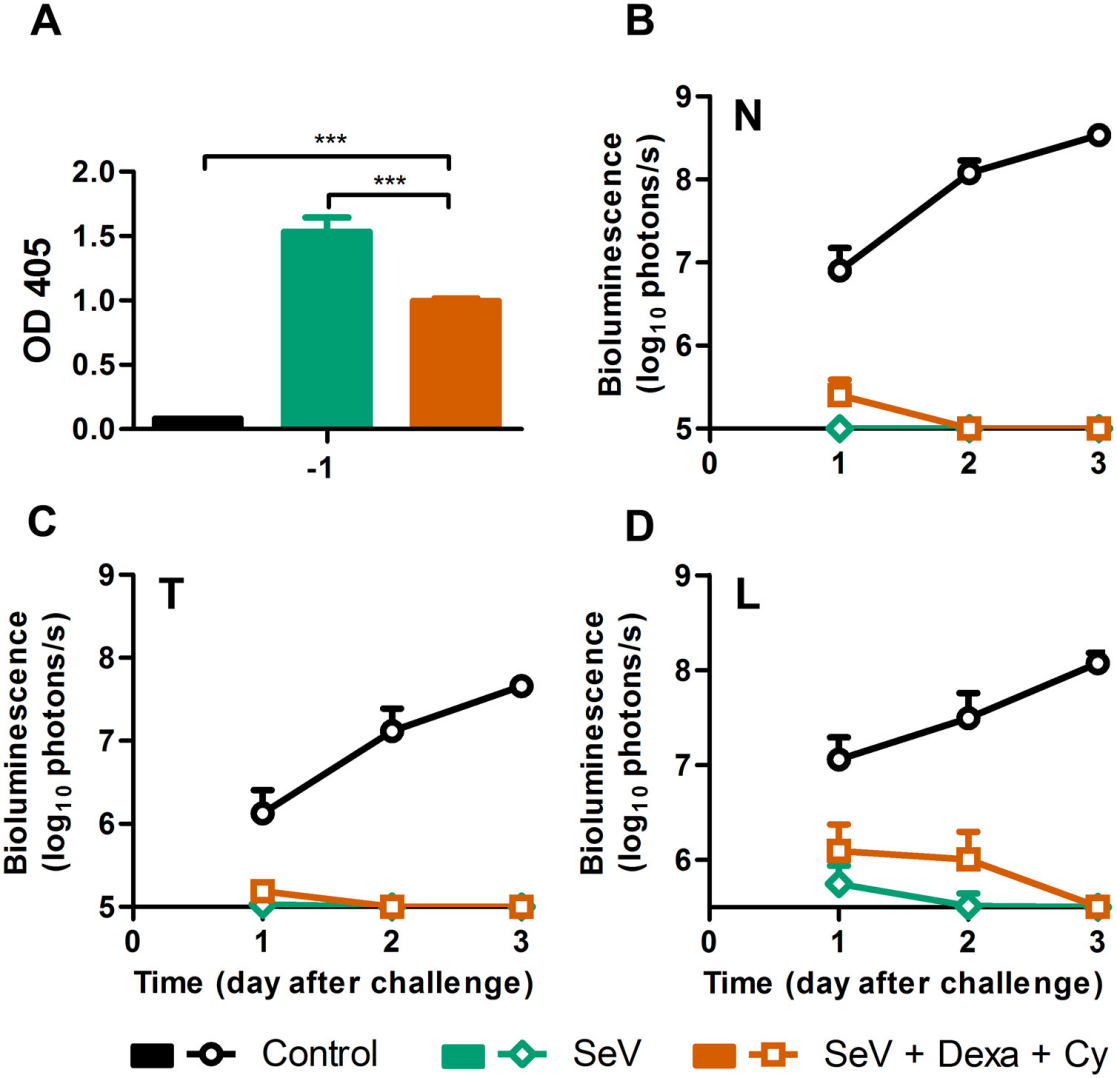
Mice infected with SeV after treatment with Dexa + Cy are protected from a secondary lethal challenge. (A) Serum SeV-specific IgG levels measured 1 day before challenge. The data are representative of 2 independent experiments with 3 mice per group. (B–D) Bioluminescence in the nasopharynx (B), trachea (C), and lungs (D) after challenge with SeV. The data are averages of 2 independent experiments with 8 mice per group. Error bars represent standard deviation. Statistics: 2-Way ANOVA; ***P < 0.001.

To assess the ability of Dexa + Cy-treated mice to mount a protective response after immunosuppression ceased and the infection had been cleared, we treated mice with Dexa and Cy, infected at 4 d.a.d.s., collected peripheral sera at 59 d.a.d.s. (55 d.p.i.), and challenged at 60 d.a.d.s. with a lethal dose (3 × 10^6^ PFU in 30 μL). Immunosuppressed infected mice seroconverted to a level lower than that observed in non-drug-treated infected mice (*P* < 0.001) (Fig 11A). Naïve control mice had high levels of infection throughout the respiratory tract (Fig 11B–11D). Naïve mice lost 20% of their starting weight while the previously immunocompromised and infected mice lost only 5% of their starting weight after challenge, significantly less than control mice (*P* < 0.01). Additionally, previously infected mice had bioluminescence levels just above the limits of detection, clearing the infection from the lungs within 3 days and from the nasopharynx and trachea within 2 days (Fig 11B–11D). Thus, while it has previously been known that prior infection provides protection from subsequent challenge in the normal host, the present work extends this finding to a pharmacologically induced immunocompromised host that becomes infected, recovers, and is then protected from a lethal challenge.

## Discussion

In this work, we studied the dynamics of SeV spread and clearance in pharmacologically immunosuppressed mice and subsequently evaluated immunotherapy strategies aimed at supplanting the components that function during the normal host response but were lacking in the immunosuppressed animals. In humans, both lymphopenia and neutropenia are associated with an increased risk or progression of parainfluenza and other respiratory viral infections to the LRT, and these conditions increase the risk of mortality [2, 10]. The contributory effect of a decrease in the lymphocyte or neutrophil count is unknown. It is unclear if increasing the neutrophil count by administering G-CSF to patients will mitigate this risk. The role of NK cells in viral clearance in unknown. The role of the antibody-mediated protective effect after intravenous immunoglobulin (Ig) is administered to patients with LRT involvement is also unclear, and it is impractical to study it in humans because of confounding variables.

Noninvasive bioluminescence imaging, used here for the first time to quantify respiratory viral dynamics in an immunocompromised host, revealed that lymphopenia and neutropenia have no discernible effects on the initial spread of infection into the LRT of mice during the first 4 days of infection; their major effect is on subsequent viral clearance. The commonly used corticosteroid Dexa delays clearance by 1 day, whereas the alkylating agent Cy delays clearance until approximately 1 week after the drug is discontinued, resulting in progressive lung infection and pathology. Severe neutropenia has been associated with progression of HPIV infection in the LRT in humans [2, 3]. Association of variables with clinical phenomena does not imply causation. Here, we provide evidence that an animal model can be invaluable in making distinctions between association and causation. Our studies revealed that SeV clearance correlates with rebounds of T lymphocytes, NK cells, and neutrophils. When using antibodies to deplete these immune cells in mice, T-cell depletion prevented, NK-cell depletion delayed, and neutrophil depletion had no apparent effect on viral clearance.

Noninvasive bioluminescence imaging, a recently developed technique, allows the infection and viral clearance to be measured throughout the respiratory tract in the same living animal over time [42, 43]. This technique has revealed that SeV and influenza virus infections are anisotropic: the mode of transmission (contact versus droplet) influences the site of infection initiation, which in turn influences viral spread and pathogenesis [20, 44]. Variola virus infections are also anisotropic [45], suggesting that this is a common feature of respiratory infections. Thus, infection dynamics should be considered when modeling respiratory viral infections.

For SeV and the related human *Respiroviruses* HPIV1 and HPIV3, contact transmission is thought to be the predominant mode of transmission [20, 46–48]. HPIV infection typically begins in the URT, first causing rhinitis and pharyngitis [16]. Severe LRT involvement in immunocompetent infants and children is relatively rare. For example, only one-quarter of HPIV1 infections cause bronchitis or pneumonia, and only approximately 3% result in severe acute croup [49, 50]. Similarly, upon contact transmission of SeV, the murine counterpart of HPIV1, the infection begins in the nasopharynx. The virus replicates to relatively high levels in the URT but is limited to a low level in the lungs of immunocompetent mice [19, 20]. We recently showed that the contact transmission dynamics of SeV can be mimicked by intranasal inoculation of virus in a 5-μL volume, whereas a 30-μl inoculation of virus directly into the lungs induces a rapid pneumonia that is uncharacteristic of natural infection [23]. High-dose inoculations (25–50 μL in mice) are commonly performed to study respiratory viral infection in immunocompromised hosts [51–54]. However, such infections lack certain clinical features associated with HPIV infections in humans, including a lag period before the virus spreads into the LRT and progressive growth in the LRT [55]. The delayed LRT infection, modeled here for SeV, represents a window of opportunity for therapeutic intervention in the immunocompromised host that would be missed by traditional studies employing high-volume inoculation techniques. This is significant, as there are currently no licensed treatments for HPIV infection.

The median time of onset for HPIV symptoms in humans is 2.6 d.p.i. [56, 57]. Here, we studied the impact of therapeutic interventions initiated after 2 days of infection with SeV in immunocompromised mice, an early time point during which the nasal infection is robust but virus growth in the lungs is nearly undetectable. We found that SeV-specific hyperimmune serum and mAbs against the SeV F and HN surface glycoproteins limited lung infection to a relatively low level. At the time of treatment, nasopharyngeal infection was already reaching a near-maximal level; as a result, antibodies were less effective at controlling nasal infection when delivered at 2 d.p.i. Although Ig administration did not reduce the high mortality resulting from HPIV infection in immunocompromised patients [12], HPIV-specific monoclonal antibodies may prove more effective at doing so. Palivizumab, an RSV-specific mAb against the viral F glycoprotein, significantly reduces hospitalization rates when used prophylactically during the RSV season in very-high-risk infants [58] and children younger than 24 months of age who are profoundly immunocompromised [59]. G-CSF, a growth factor that stimulates neutrophils, has been shown to decrease morbidity and mortality from infections in randomized controlled trials when used prophylactically after chemotherapy [60–62] and may enhance the clearance of influenza virus infection by neutrophil-mediated guidance and maintenance of CD8^+^ T cells [31, 32]. A lack of G-CSF increases morbidity and mortality due to Sendai and influenza viruses infections [36]. In our model, therapeutic G-CSF and GM-CSF had no apparent effect on SeV infection.

Depleting NK cells substantially delayed viral clearance; however, mice were still capable of clearing the infection albeit with an increase in weight loss. NK cells have been shown previously to play a role during influenza virus infection [63] and to regulate the functions of T lymphocytes during HPIV3 infection [64]. Here we showed NK cells play a role in viral clearance after recovery from immunosuppressive drug treatment. Our data also show that depleting T cells leads to chronic infection, despite a 2.8 fold increase in the absolute numbers of NK cells (1370.36 ± 416 versus 494.5 ± 333.2 cells/ μL). This indicates that NK cells are necessary for efficient clearance of infection but are not sufficient for clearance, at least in the absence of B- and T-cell responses. As antibody dependent cell mediated cytotoxicity (ADCC) is a major mechanism by which NK cells help clear virally infected cells [65], it will be of interest to test a possible therapeutic potential of NK cells in clearing viral infection with concurrent administration of monoclonal antibodies. This approach is currently clinically used for treatment of cancer [66]. The immunocompromised model of SeV infection developed here is well suited for preclinical testing of other therapeutic agents. DAS181, a novel sialidase fusion protein active against parainfluenza virus [67, 68] is currently undergoing a randomized placebo-controlled clinical trial in immunosuppressed older children and adults (NCT01644877). Our mouse model could provide insights into the efficacy of this drug in preventing progression to LRT disease, which is associated with high morbidity.

Dexa and Cy have different mechanisms of action that have complementary immunosuppressive effects. Dexa causes apoptosis of lymphocytes and malignant cells when administered daily in pulses, which are needed to saturate the glucocorticoid receptor [25]. Dexa is commonly given as pulse therapy against acute lymphoblastic leukemia, lymphoma, and Hodgkin’s disease [15]. Together, these diseases constitute a quarter of all malignant neoplasms in children. Cy, a potent immunosuppressive drug, is a myelosuppressive agent that exerts its cytotoxic effect by binding to DNA, resulting in apoptosis. Cy is used to treat aplastic anemia [69] and to prevent graft-versus-host disease in recipients of allogeneic HSCT [70]. Myelosuppression is the major dose-limiting toxicity affecting lymphocytes, neutrophils, red blood cells, and platelets. In contrast to Dexa, which increases the neutrophil count, Cy causes a dose-dependent suppression of neutrophils. The two drugs are often given in combination to treat hematologic malignant disease [71, 72].

The recovery of immune function and clearance of HPIV infection in humans after Dexa pulse therapy, alone or in combination with Cy, has not been studied but has important clinical implications. Patients who have leukemia or are in remission may contract an HPIV infection either before scheduled chemotherapy or while receiving chemotherapy, and this may delay viral clearance. Understanding the temporal sequence of disease progression, clearance, and chemotherapy and its relationship to lymphocyte and neutrophil recovery is critical to making decisions on whether to delay chemotherapy or allow for immune recovery before subsequent courses of treatment. This will balance the risk of morbidity and mortality from infection against the risk of leukemia recurrence as a result of decreasing the dose-intensity of chemotherapy.

In the mouse model, we found that Dexa monotherapy delayed SeV clearance by 1 day and did not result in a severe pulmonary infection. If similar results could be achieved with HPIV infections in humans, the minor benefit derived from delaying scheduled Dexa chemotherapy would most likely be outweighed by the risk of leukemia recurrence, supporting continuation of Dexa treatment clinically after onset of PIV infection. The relatively mild effects of Dexa treatment during SeV infection may be virus-family specific or model dependent, as influenza and Enterovirus 71 infections in mice have been associated with substantially increased viral titers [73, 74]. In contrast to Dexa, Cy therapy in mice led to a persistent SeV infection with increasing LRT involvement that began to clear one week after the drug was discontinued. Thus, a SeV infection that began before the start of treatment lasted longer than an infection initiated later; in both cases, they cleared at the same time. If the same pattern occurs with HPIV in humans, the increased risk of a lung infection that is prolonged and progressive would suggest that there is an overall benefit in delaying Cy treatment until the HPIV infection is controlled.

Overall, this work shows that noninvasive imaging both enhances the clarity with which prolonged infection can be followed and provides an innovative tool for dissecting host-pathogen interactions and evaluating therapeutic options in an animal model system. Our major findings include the following: (a) defining the kinetics of the delay in viral clearance caused by the chemotherapeutic agents Dexa and Cy, (b) associating clearance with T lymphocyte and NK cell (but not neutrophil) rebound, (c) elucidating the mechanism of clearance by immune-cell depletion studies, (d) showing that the memory response to reinfection is preserved even after chemotherapy, and (e) demonstrating the benefit of therapeutic mAbs (but not of G-CSF and GM-CSF) in the immunocompromised host. This model may serve as a platform for evaluating responses to other drugs and vaccines, and similar models could be established for other luciferase-expressing respiratory viruses, such as influenza virus [75–77].

## Materials and Methods

### Ethics statement

All animal studies were conducted following the Guide for the Care and Use of Laboratory Animals [78] from the National Research Council of the National Academies of the USA. Mice experiments were approved by the Animal Care and Use Committee of St. Jude Children’s Research Hospital (protocol number 459) and were performed in compliance with relevant institutional policies, the Association for the Accreditation of Laboratory Animal Care guidelines, the National Institutes of Health regulations, and local, state, and federal laws. Animals were anesthetized with isoflurane and euthanized by CO2.

### Drugs, antibodies, and reagents

Dexa and Cy were purchased from Sigma-Aldrich (St. Louis, MO). Dexa was administered i.p. in 10 daily doses, each of 10 mg/kg/day in 200 μL. Cy was administered i.p. in 2 doses of 150 mg/kg in 300 μL. The anti-Ly6G clone 1A8 was purchased from BioXcell (West Lebanon, NH) and administered i.p. in 3 doses, each of 0.5 mg in 100 μL. The rhG-CSF (NEUPOGEN; Amgen Inc., Thousand Oaks, CA) was administered i.p. in 7 doses, each of 125 μg/kg in 200 μL. The recombinant murine GM-CSF (Peprotech, catalog no. 315–03) was administered either intranasally in a dose of 100 ng/ mouse in 10 μL PBS or i.p. in a dose of 200 ng/ mouse in 100 μL PBS in 7 doses starting at 6 d.a.d.s. The anti-mouse CD3e clone 145-2C11 (catalog no. BP0001-1) was purchased from BioXcell and was given in two doses of 20 μg each i.p. in 100 μL PBS at days 11 and 14. The Armenian Hamster IgG was used as an isotype control and was purchased from BioXcell (catalog no. BP0091). Anti asialo GM1 antibody was purchased from Wako Chemicals (Richmond, VA) and each mouse was given 25 μL of the antibody in a total of 100 μL volume i.p. Normal rabbit serum was used as a control (MP Biomedicals, catalog no. 191357). Mouse IgG was purchased from BioXcell (catalog no. BE0093) and administered i.p. in a single dose of 230 μg. The SeV mAbs S2 and M16 were purified from ascites fluid by using protein G columns (GE Healthcare, catalog no. 17-0404-01). Briefly, ascites fluid was treated overnight at 4°C with PHM-L Liposorb absorbent resin (Calbiochem, catalog no. 524371) at a ratio of 1 g resin to 25 mL ascites. The ascites fluid was then centrifuged at 1000 × g for 5 min, transferred to a new tube, diluted 1:10 with 1× PBS, and filtered through a 0.2-μm filter. The diluted fluid was passed through the protein G column by using a 10-mL syringe after washing the column 5 times with water and 5 times with 1× PBS. The column was washed until the optical density at 280 nm (A280) of the flow-through was less than 0.002. The antibody was eluted with 5 mL of 0.1 M glycine-HCL solution, pH 2.7, and 1-mL fractions were collected in tubes that already contained 100 μL of 1 M Tris-HCL, pH 8. The antibody concentrations in each fraction were determined by measuring the A280. To desalt the antibody, Zeba desalting columns (ThermoFischer, catalog no. 89889) were used according to the manufacturer’s protocol.

### Bioluminescence imaging of mice

Eight-week-old BALB/c mice (Jackson Laboratory) were infected with rSeV-luc(M-F*), a recombinant Sendai virus of the Enders strain, which was constructed and described previously [19]. For the infections, mice were anesthetized with isoflurane and inoculated intranasally with 5 μL of PBS or PBS containing 7,000 PFU of the virus. The animals were monitored daily for weight loss, morbidity, and mortality. Before the mice were imaged, they were i.p. injected with D-luciferin (PerkinElmer, catalog no. 122796) at a dose of 150 mg/kg of body weight in a volume of 200 μL and anesthetized with isoflurane for 5 min. *In vivo* images were acquired with a Xenogen IVIS CCD camera system (Caliper Life Sciences) and analyzed with Living Image 4.5 software (Caliper Life Sciences). To quantify the bioluminescence, regions of interest (ROI) were defined manually, as described previously [19], and the data were expressed as total flux (photons/s).

### Tissue collection for viral titration and pathologic analysis

Mice were euthanized using CO2, then tissue samples for viral titration were collected in 500 μL PBS supplemented with 1% penicillin and 1% streptomycin, homogenized with a TissueLyser II (Qiagen), and centrifuged. The supernatants were collected and titered on LLC-MK2 monkey kidney epithelial cells (ATCC CCL-7), maintained in Dulbecco’s modified Eagle’s medium supplemented with 10% FBS, by a standard plaque assay as described previously [79]. For pathologic analysis, nasal cavities, tracheas, and lungs were collected in 10% neutral buffered formalin. Paraffin embedding, sectioning, and staining of tissues were performed by the Veterinary Pathology Core Laboratory at St. Jude Children’s Research Hospital.

### Blood, bronchoalveolar lavage, and spleen collection and cellular counts

For blood collection, mice were anesthetized with isoflurane and blood was collected in 10% EDTA (disodium) via the retro-orbital route. The cells were counted with an automatic cell counter. For BALF collection, mice were euthanized, then their tracheas were intubated and washed 3 times with 0.5 mL of PBS containing 2 mM EDTA, giving a total lavage volume of 1.5 mL. The BALF was centrifuged, the supernatants were collected for ELISA and cytokine analysis (using a Millipore MILLIPLEX Mouse Cytokine/Chemokine Magnetic Bead Panel), and the cellular pellet was resuspended in 200 μL PBS and the cells were counted with an automatic cell counter. The 32 cytokines and chemokines were the following: Eotaxin/CCL11, G-CSF, GM-CSF, IFN-γ, IL-1α, IL-1β, IL-2, IL-3, IL-4, IL-5, IL-6, IL-7, IL-9, IL-10, IL-12 (p40), IL-12 (p70), IL-13, IL-15, IL-17, IP-10, KC-like, LIF, LIX, MCP-1, M-CSF, MIG, MIP-1α, MIP-1β, MIP-2, RANTES, TNF-α, and VEGF. For spleen collection, mice were euthanized and their spleens were harvested, weighed, passed through 70-μm cell strainers (BD Falcon), washed with PBS, passed again through 70-μm cell strainers, and centrifuged. The spleen cells were then suspended in equal volumes of PBS and counted with an automatic cell counter.

### ELISA

ELISA was performed as described previously [19], except that goat anti-mouse IgG conjugated to alkaline phosphatase (AP) (Southern Biotechnology) was used, then p-nitrophenyl phosphate substrate was added to the wells (Sigma) and the plates were read at a wavelength of 405 nm. BALF samples for ELISA were not diluted. For serum ELISA, the samples were serially diluted; the data shown are for samples diluted 1:10.

### Flow cytometry

Blood cells, collected as described earlier, were blocked in mouse BD Fc block (BD Biosciences, catalog no. 553141) in staining medium (PBS + 0.5% FBS) for 10 minutes at 4°C, then stained with CD3-APC, CD4-FITC, CD8-PE, CD19-PE-Cy7, CD49b-APC-Cy7 antibodies (BD Biosciences) for 30 minutes at 4°C. Red blood cells were lysed using BD FACSTM lysing solution (catalog no. 349202). Cellular populations were gated and quantified by using Flowjo version 7.6.1.

### Preparation of SeV-specific hyperimmune sera

BALB/C mice were infected or mock infected at 10^5^ PFU in a volume of 30 μL with wild-type SeV, Enders strain, and were challenged with the same dose of virus 3 weeks later. At day 45 after the initial infection, blood was collected and sera were obtained after centrifuging the coagulated blood and pooled. SeV-specific antibody titers were determined by ELISA, and every mouse was injected with 200 μL of the control or hyperimmune serum.

### Statistical analyses

Statistical analyses were performed using GraphPad prism 5. Student’s *t*-test and 2-way analysis of variance (ANOVA) followed by Bonferroni post-tests were used to test the differences between groups. *P*-values of less than 0.05 were considered significant.

## Supporting Information

S1 Fig Effect of Dexa and/or Cy on peripheral blood lymphocytes and neutrophils. (A) Drug treatment regimen. Arrows denote days on which drug injections were performed. (B) Percent change in body weight after starting drug treatment. (C) Peripheral blood lymphocyte counts. (D) Peripheral blood neutrophil counts. The data are averages of 5 mice per group. Groups include PBS (black circles), Dexa (green diamonds), Cy (orange squares), and Dexa + Cy (blue triangles). Error bars represent the standard deviation.
(TIF)

S2 Fig Effect of Dexa and/or Cy on the spleen. Groups of mice were euthanized at the reported time points to recover spleens and measure splenic weight (A), splenic lymphocytes (B), and splenic neutrophils (C). Groups include PBS (black circles or bars), Dexa (green diamonds or bars), Cy (orange squares or bars), and Dexa + Cy (blue triangles or bars). The data are averages of 5 mice per group. Error bars represent the standard deviation.
(TIF)

S3 Fig Bioluminescence imaging of SeV inoculated into mice 4 days after treatment started. Groups of mice were treated with (A) PBS, (B) Dexa, (C) Cy, or (D) Dexa + Cy. Bioluminescence is reported for the nasopharynx (red triangles), trachea (orange circles), and lungs (blue squares). The data shown are averages of 3 independent experiments with 15 mice in each group. d.p.i., days postinfection; d.a.d.s., days after drug started.
(TIF)

S4 Fig Timing of SeV spread and clearance as a function of inoculation time point. (A–C) Comparison of the kinetics of viral spread in mice infected 1 day before (lighter colors) or 4 days after (darker colors) starting treatment with (A) Dexa, (B) Cy, or (C) Dexa + Cy. (D) Progression of SeV infection when Cy was given in 4 doses 5 days apart on 0, 5, 10, and 15 d.a.d.s. Symbols denote the following treatment groups: PBS (black circles), Dexa (green diamonds), Cy (orange squares), and Dexa + Cy (blue triangles). The data shown are the average bioluminescence of 5 mice per group at each time point. N, nasal; T, trachea; L, lungs.
(TIF)

S5 Fig Histopathology in nasal cavities. Groups of mice were inoculated with SeV 4 days after Dexa + Cy (or PBS) treatment started and euthanized 6 days postinfection (10 d.a.d.s.) (A) or 12 days postinfection (16 d.a.d.s.) (B) so the nasal cavities could be fixed, stained, and analyzed by microscopy. Sections were stained with hematoxylin and eosin (H&E) (left panels), with a mAb to CD3 to show T-cell infiltration (middle panels), or with a mAb to SeV (right panels). Sections from Dexa + Cy—treated mice (bottom panels) were compared to sections from untreated controls (upper panels). The data are representative of the 4 different animals in each group.
(TIF)

S6 Fig Effect of GM-CSF on viral clearance. (A–C) Bioluminescence in the nasopharynx (A), trachea (B), and lungs (C) after administering GM-CSF or PBS intranasally in a dose of 100 ng/ mouse starting at 6 d.a.d.s. for 7 doses. Dexa and Cy injections were performed as described previously, and 7000 PFU SeV was intranasally inoculated in 5 μL PBS at 4 d.a.d.s. (D-F) Neutrophil (D), lymphocyte (E), and monocyte (F) counts in the BALF collected at 14 d.a.d.s. (G) Concentration of IP-10 in the BALF collected at 14 d.a.d.s. (H-J) Bioluminescence in the nasopharynx (H), trachea (I), and lungs (J) after treating with GM-CSF i.p. with 7 doses of 200 ng/mouse starting at 6 d.a.d.s. Dexa and Cy injections were performed as described previously, and 7000 PFU SeV was intranasally inoculated in 5 μL PBS at 4 d.a.d.s. Groups include PBS (black bars and circles), Dexa + Cy (light blue bars and triangles), Dexa + Cy + GM-CSF (green bars and rectangles), and Dexa + Cy + control intranasal PBS (gray bars and diamonds). The data shown are averages of 5 mice per group. In all graphs, error bars represent the standard deviation. d.p.i., days postinfection; d.a.d.s., days after drug started. * *P* < 0.05, ** *P* < 0.01*** *P* < 0.001.
(TIF)
