# Supplementary figures and images for: Non-invasive Imaging of Sendai Virus Infection in Pharmacologically Immunocompromised Mice: NK and T Cells, but not Neutrophils, Promote Viral Clearance after Therapy with Cyclophosphamide and Dexamethasone
Source: PLoS Pathog. 2016 Sep 2;12(9):e1005875. doi: 10.1371/journal.ppat.1005875 (PMC5010285; doi:10.1371/journal.ppat.1005875)

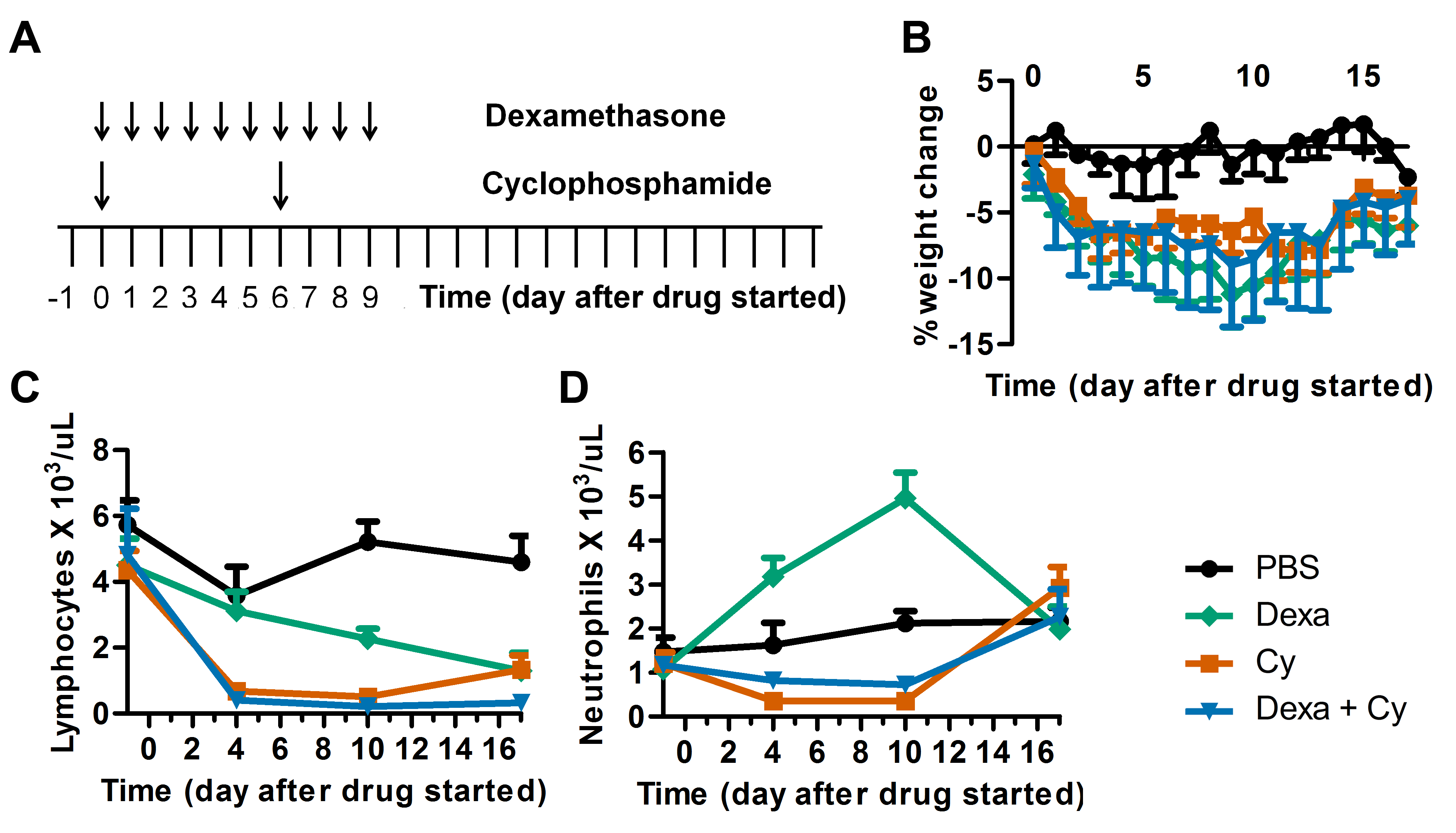

Supplement: S1 Fig — (A) Drug treatment regimen. Arrows denote days on which drug injections were performed. (B) Percent change in body weight after starting drug treatment. (C) Peripheral blood lymphocyte counts. (D) Peripheral blood neutrophil counts. The data are averages of 5 mice per group. Groups include PBS (black circles), Dexa (green diamonds), Cy (orange squares), and Dexa + Cy (blue triangles). Error bars represent the standard deviation. (TIF) [file ppat.1005875.s001.tif]

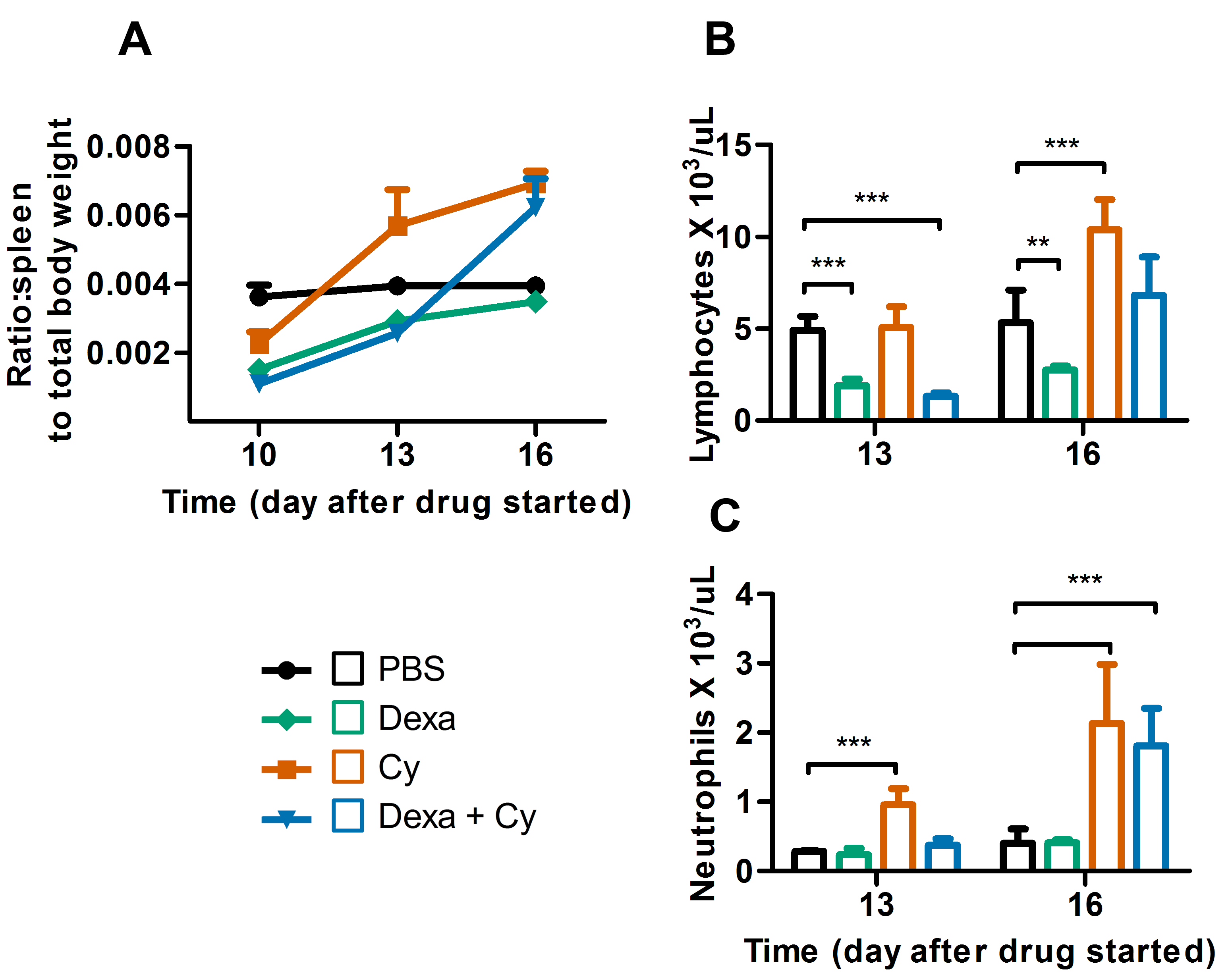

Supplement: S2 Fig — Groups of mice were euthanized at the reported time points to recover spleens and measure splenic weight (A), splenic lymphocytes (B), and splenic neutrophils (C). Groups include PBS (black circles or bars), Dexa (green diamonds or bars), Cy (orange squares or bars), and Dexa + Cy (blue triangles or bars). The data are averages of 5 mice per group. Error bars represent the standard deviation. (TIF) [file ppat.1005875.s002.tif]

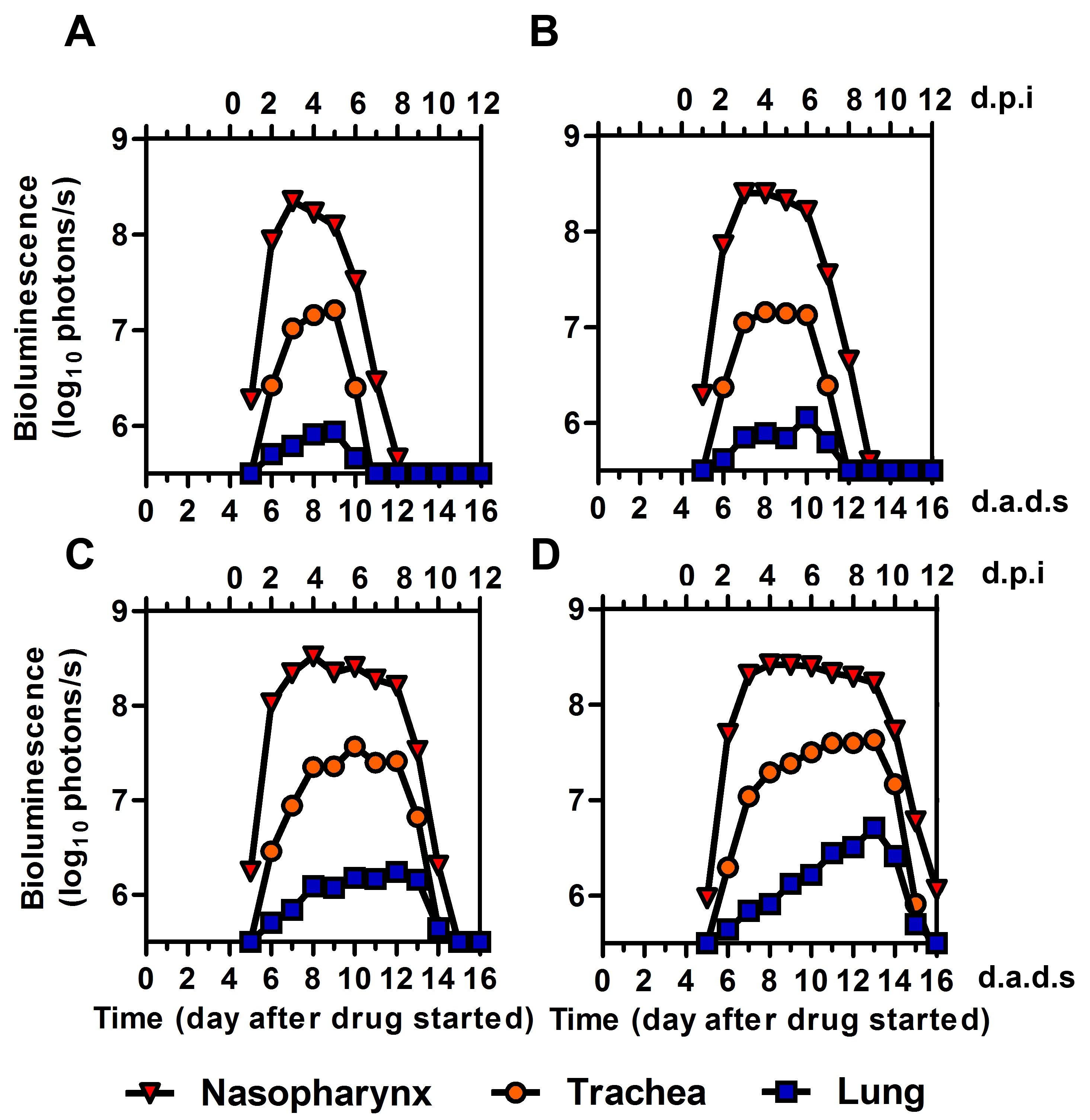

Supplement: S3 Fig — Groups of mice were treated with (A) PBS, (B) Dexa, (C) Cy, or (D) Dexa + Cy. Bioluminescence is reported for the nasopharynx (red triangles), trachea (orange circles), and lungs (blue squares). The data shown are averages of 3 independent experiments with 15 mice in each group. d.p.i., days postinfection; d.a.d.s., days after drug started. (TIF) [file ppat.1005875.s003.tif]

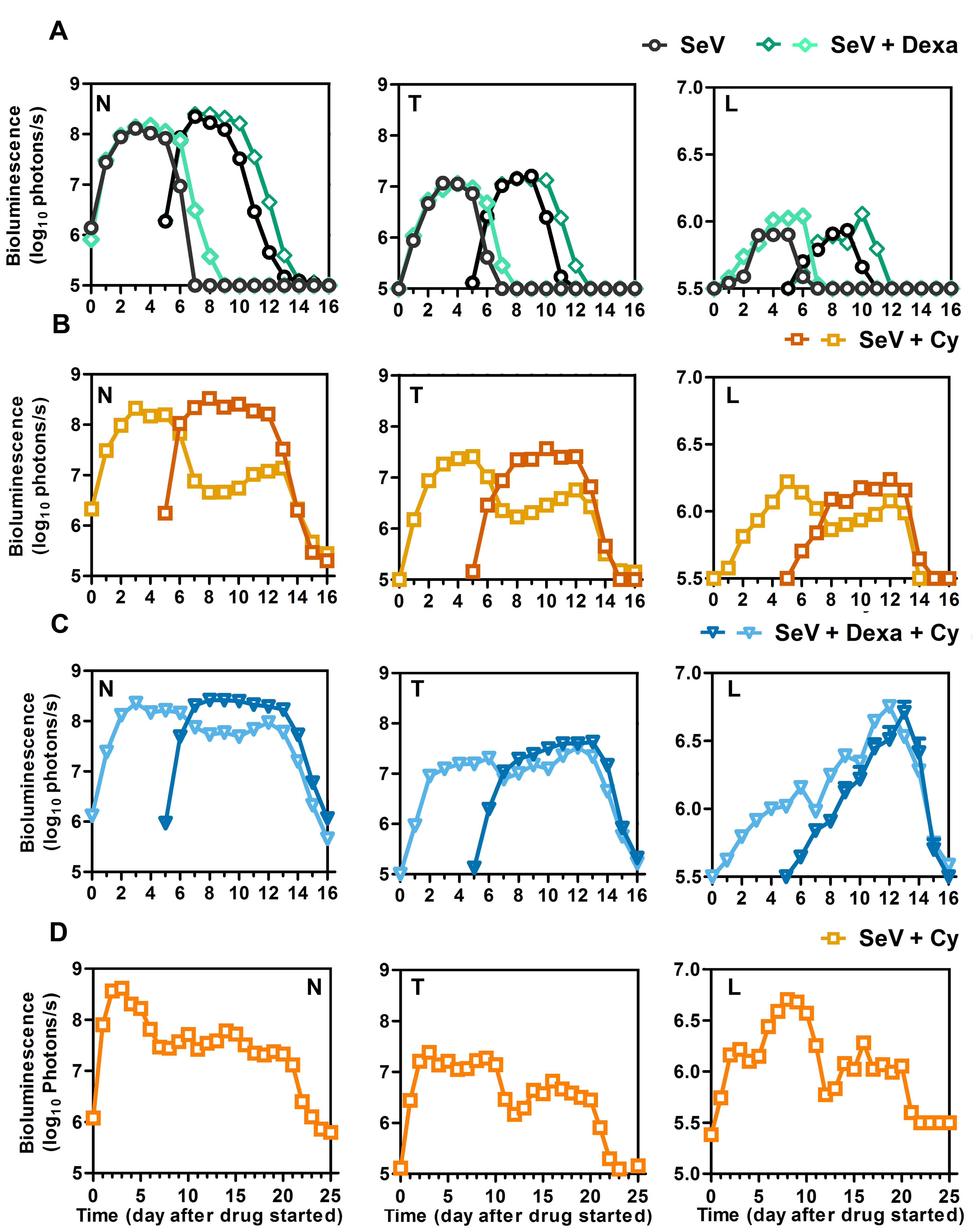

Supplement: S4 Fig — (A–C) Comparison of the kinetics of viral spread in mice infected 1 day before (lighter colors) or 4 days after (darker colors) starting treatment with (A) Dexa, (B) Cy, or (C) Dexa + Cy. (D) Progression of SeV infection when Cy was given in 4 doses 5 days apart on 0, 5, 10, and 15 d.a.d.s. Symbols denote the following treatment groups: PBS (black circles), Dexa (green diamonds), Cy (orange squares), and Dexa + Cy (blue triangles). The data shown are the average bioluminescence of 5 mice per group at each time point. N, nasal; T, trachea; L, lungs. (TIF) [file ppat.1005875.s004.tif]

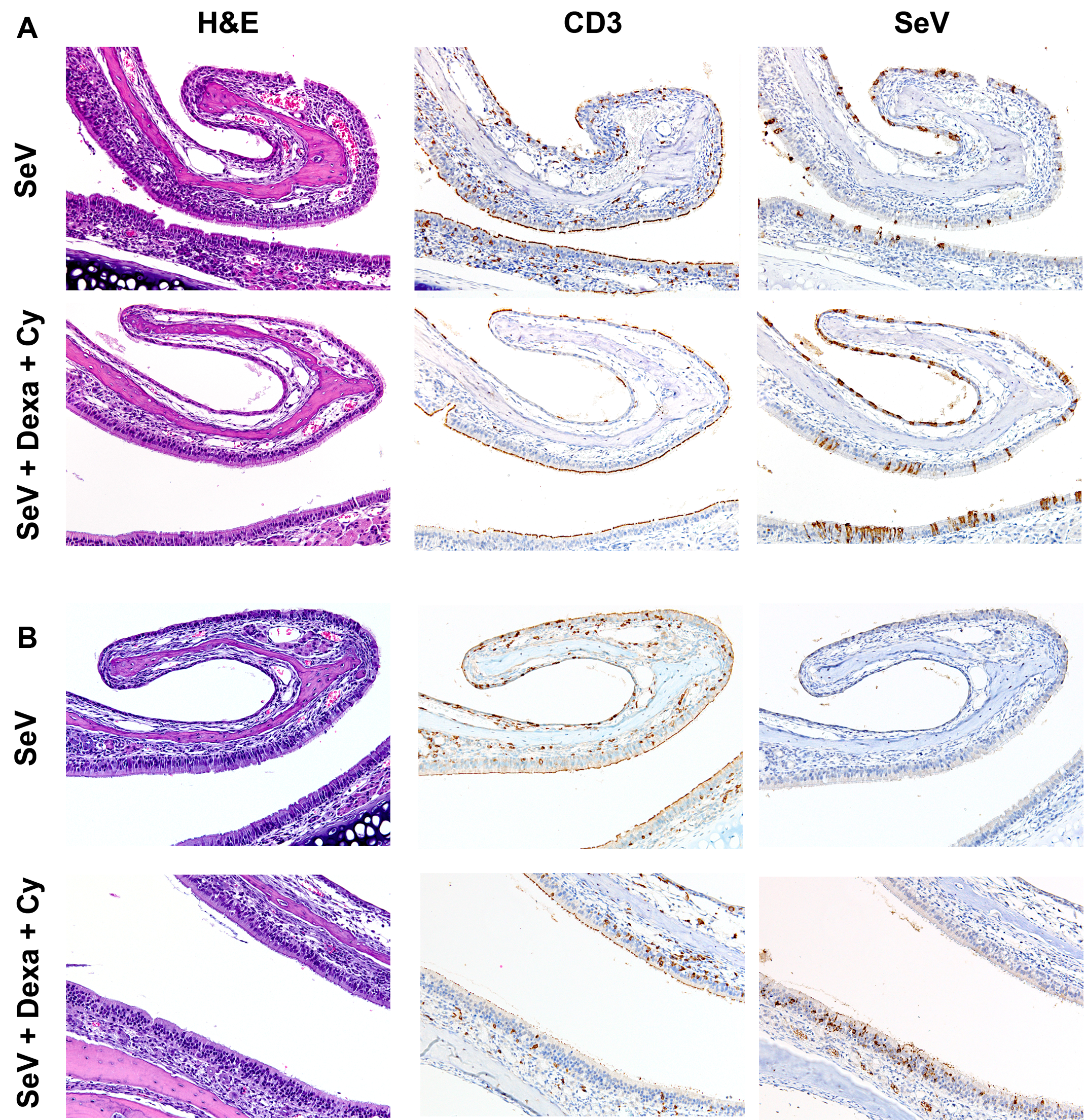

Supplement: S5 Fig — Groups of mice were inoculated with SeV 4 days after Dexa + Cy (or PBS) treatment started and euthanized 6 days postinfection (10 d.a.d.s.) (A) or 12 days postinfection (16 d.a.d.s.) (B) so the nasal cavities could be fixed, stained, and analyzed by microscopy. Sections were stained with hematoxylin and eosin (H&E) (left panels), with a mAb to CD3 to show T-cell infiltration (middle panels), or with a mAb to SeV (right panels). Sections from Dexa + Cy—treated mice (bottom panels) were compared to sections from untreated controls (upper panels). The data are representative of the 4 different animals in each group. (TIF) [file ppat.1005875.s005.tif]

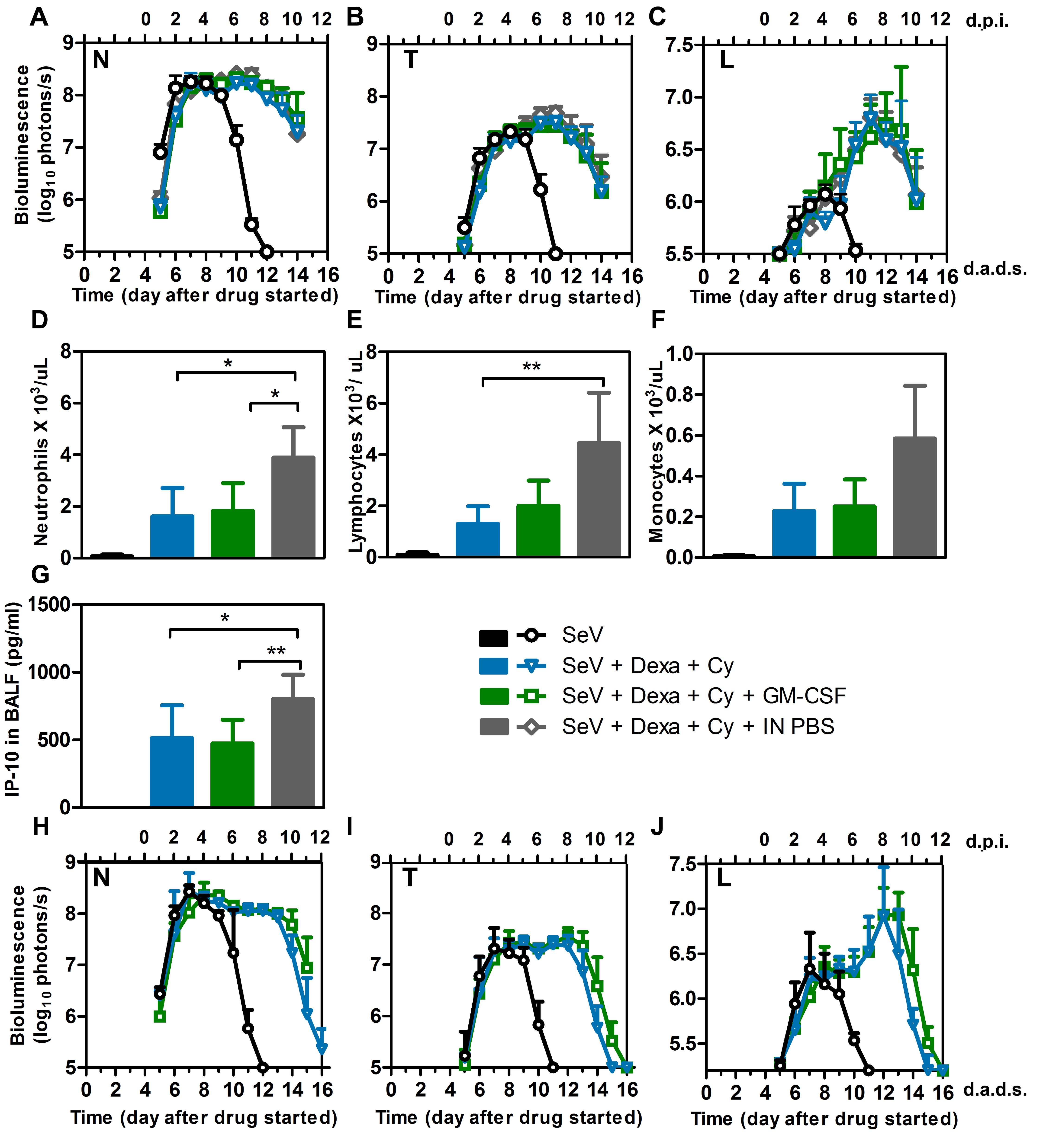

Supplement: S6 Fig — (A–C) Bioluminescence in the nasopharynx (A), trachea (B), and lungs (C) after administering GM-CSF or PBS intranasally in a dose of 100 ng/ mouse starting at 6 d.a.d.s. for 7 doses. Dexa and Cy injections were performed as described previously, and 7000 PFU SeV was intranasally inoculated in 5 μL PBS at 4 d.a.d.s. (D-F) Neutrophil (D), lymphocyte (E), and monocyte (F) counts in the BALF collected at 14 d.a.d.s. (G) Concentration of IP-10 in the BALF collected at 14 d.a.d.s. (H-J) Bioluminescence in the nasopharynx (H), trachea (I), and lungs (J) after treating with GM-CSF i.p. with 7 doses of 200 ng/mouse starting at 6 d.a.d.s. Dexa and Cy injections were performed as described previously, and 7000 PFU SeV was intranasally inoculated in 5 μL PBS at 4 d.a.d.s. Groups include PBS (black bars and circles), Dexa + Cy (light blue bars and triangles), Dexa + Cy + GM-CSF (green bars and rectangles), and Dexa + Cy + control intranasal PBS (gray bars and diamonds). The data shown are averages of 5 mice per group. In all graphs, error bars represent the standard deviation. d.p.i., days postinfection; d.a.d.s., days after drug started. * P < 0.05, ** P < 0.01*** P < 0.001. (TIF) [file ppat.1005875.s006.tif]
